# UTexas Aptamer Database: the collection and long-term preservation of aptamer sequence information

**DOI:** 10.1093/nar/gkad959

**Published:** 2023-10-30

**Authors:** Ali Askari, Sumedha Kota, Hailey Ferrell, Shriya Swamy, Kayla S Goodman, Christine C Okoro, Isaiah C Spruell Crenshaw, Daniela K Hernandez, Taylor E Oliphant, Akshata A Badrayani, Andrew D Ellington, Gwendolyn M Stovall

**Affiliations:** Freshman Research Initiative, The University of Texas, Austin, TX 78712, USA; Freshman Research Initiative, The University of Texas, Austin, TX 78712, USA; Freshman Research Initiative, The University of Texas, Austin, TX 78712, USA; Freshman Research Initiative, The University of Texas, Austin, TX 78712, USA; Freshman Research Initiative, The University of Texas, Austin, TX 78712, USA; Freshman Research Initiative, The University of Texas, Austin, TX 78712, USA; Freshman Research Initiative, The University of Texas, Austin, TX 78712, USA; Freshman Research Initiative, The University of Texas, Austin, TX 78712, USA; Freshman Research Initiative, The University of Texas, Austin, TX 78712, USA; Freshman Research Initiative, The University of Texas, Austin, TX 78712, USA; Institute for Molecular Biosciences, The University of Texas, Austin, TX 78712, USA; Center for Systems and Synthetic Biology, The University of Texas, Austin, TX 78712, USA; Freshman Research Initiative, The University of Texas, Austin, TX 78712, USA; Institute for Molecular Biosciences, The University of Texas, Austin, TX 78712, USA; High School Research Initiative, The University of Texas, Austin, TX 78712, USA

## Abstract

A growing interest in aptamer research, as evidenced by the increase in aptamer publications over the years, has led to calls for a go-to site for aptamer information. A comprehensive, publicly available aptamer dataset, which may be a repository for aptamer data, standardize aptamer reporting, and generate opportunities to expand current research in the field, could meet such a demand. There have been several attempts to create aptamer databases; however, most have been abandoned or removed entirely from public view. Inspired by previous efforts, we have published the UTexas Aptamer Database, https://sites.utexas.edu/aptamerdatabase, which includes a publicly available aptamer dataset and a searchable database containing a subset of all aptamer data collected to date (1990–2022). The dataset contains aptamer sequences, binding and selection information. The information is regularly reviewed internally to ensure accuracy and consistency across all entries. To support the continued curation and review of aptamer sequence information, we have implemented sustaining mechanisms, including researcher training protocols, an aptamer submission form, data stored separately from the database platform, and a growing team of researchers committed to updating the database. Currently, the UTexas Aptamer Database is the largest in terms of the number of aptamer sequences with 1,443 internally reviewed aptamer records.

## Graphical Abstract

**Graphical Abstract:**
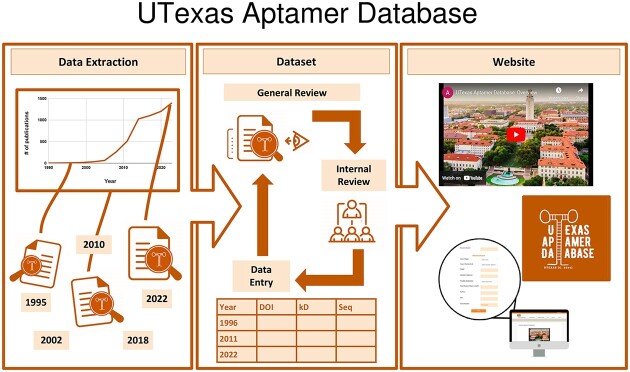

## Introduction

In the last 30 years, there has been an exponential growth of aptamer publications, with roughly 19 000 aptamer papers indexed in Pubmed (2023). There is a growing interest in collecting aptamer sequence data in a centralized repository to, for instance, browse available aptamers and applications, mine aptamer sequences for trends and improve aptamer selections (1–6). A publicly available aptamer dataset could meet that need and standardize the reporting of aptamer sequences and nucleic acid pool, aggregate data and expand on current research in the aptamer field.

Aptamers, which are similar to antibodies but are instead comprised of oligonucleotides, selectively bind to specific target molecules (e.g. proteins, carbohydrates, cells, etc.) and are used for a variety of therapeutic, diagnostic and sensor applications. Synthetically-derived aptamers are selected via a process termed SELEX (Systematic Evolution of Ligands by Exponential enrichment). Beginning with a random oligonucleotide library or pool, sieving for target-binding oligonucleotide sequences and then amplifying those target-bound oligonucleotide sequences, the pool for target-binding sequences is enriched after multiple rounds of selection (typically 5–20+ rounds) (7,8).

There is a growing need for the compilation and organization of aptamer sequences and selection information to offset some of the recent challenges within the field. To this point, Miller *et al.* traced the use of popular aptamers through the literature and, after reviewing hundreds of aptamer papers, they found that 41% of the papers contained unexplained aptamer sequence changes or omitted the sequence completely. Their work highlighted the difficulty in extracting sequence information from figures, as well as noted that many of the reported aptamer sequences contained deletions (such as the deletion of the 5′ and/or 3′ static regions), substitutions and additions, among other unexplained changes from the original aptamer sequence (2). In this context, Baird implied the need to communicate aptamer research information and highlighted the need for aptamers against a variety of targets. In this, he coined the phrase ‘thrombin problem,’ which describes the use of the same aptamer by many researchers instead of the selection and use of new aptamers and those against more clinically relevant targets (9). Given these challenges, an aptamer database would help researchers in verifying aptamer sequence information, as well as serve as a helpful resource to ascertain what aptamers currently exist and the variety of targets they bind, all collectively serving the aptamer research community and encouraging future research.

In this work, we created a publicly accessible aptamer dataset and searchable database, (https://sites.utexas.edu/aptamerdatabase), which includes publications spanning decades. With sustainability in mind, the UTexas Aptamer Database project provides access to both the raw aptamer dataset as well as the searchable database, opportunities for aptamer entry feedback, as well as several details critical to replicate aptamer work. Further, by integrating the work into a university course-based research experience, the database may be maintained and regularly updated. Ultimately, The UTexas Aptamer Database aims to collect, preserve and maintain aptamer research, thereby eliminating unnecessary barriers for new researchers, and generating a greater understanding of aptamer research for the advancement of the field overall.

## Material and methods

**Figure 1.**
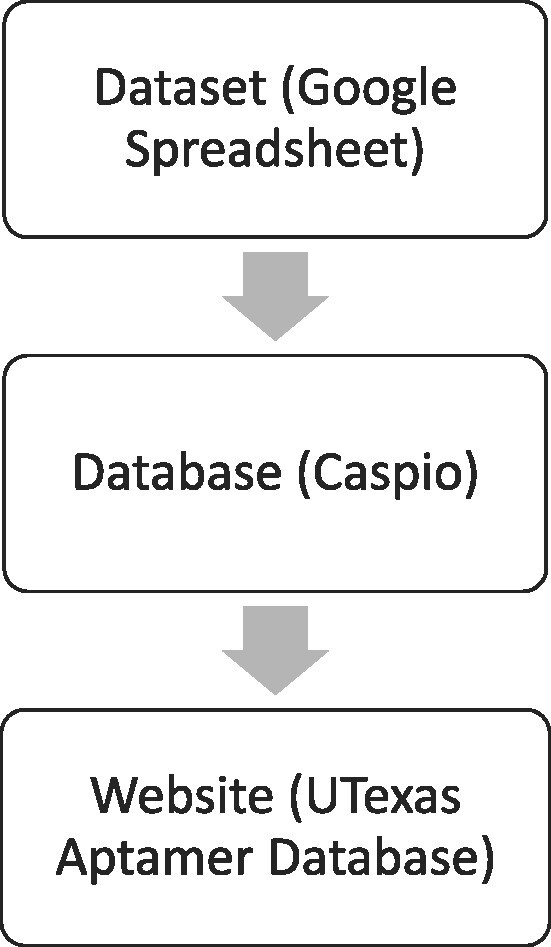
Flow of information processing in UTexas Aptamer database.

Through this research, we generated a methodology for the collection of aptamer sequences and related information, as well as constructed a publicly accessible aptamer dataset, database and website. Here we describe the methodology to construct the aptamer dataset and critical design details in the construction of the database. The Google Sheets dataset includes aptamer sequences and related information in detail (see Table 1 for information categories and Table 2 for the list of aptamer nucleic acid modifications included to date). The Database organizes and queries the dataset, offering users an interactive experience with aptamer information. Lastly, the website increases the visibility of the dataset/database while providing supporting information and best practices (see Figure 1). Collectively, all three pieces of this project are referred to as the UTexas Aptamer Database.

**Table 1.** Data reported in the UTexas Aptamer Database for each aptamer record

Database categories	Description (range of responses)
**Year of Publication**	Year of the published manuscript, currently contains manuscripts from the years 1990 to 2022
**DOI**	Digital Object Identifier (DOI) of the published manuscript
**Citation**	APA formatted citation
**Nucleic Acid**	ssRNA, ssDNA, Modified RNA, etc. Refer to Table 3 for the full list of nucleic acid modifications
**Name of Aptamer**	Name of the aptamer sequence as it is referred to in the publication (e.g. 26A-t, 13A, ligand 13, etc.)
**Target**	The full name and abbreviated name of the aptamer target, e.g. Vascular Endothelial Growth Factor (VEGF)
**Aptamer Sequence**	Includes the full sequence of the aptamer, such as the 5′constant region-random region-3′constant region, if appropriate
**GC Content**	Total % of guanines and cytosines (%GC) of the aptamer sequence (currently ranges from 28%-84%)
**Sequence Length**	The aptamer sequence length in nucleotides (nt). Lengths currently range from 6 nt to 169 nt or, in the case of chimeras (multi-aptamer or oligonucleotide construct), up to 317 nt
**Serial Number**	A serial number is assigned by our group to each aptamer with the XXX,XXX,XXX format
**Binding Affinity**	The *K*_d_ or relative *K*_d_ of the aptamer for its target, typically reported in nM concentration
**Binding/ Selection Buffer**	The binding buffer, monovalent salts (if any) and divalent salts (if any), and pH, are reported. If there was some uncertainty as to the original buffer used, then that was noted (e.g. ‘Binding buffer for assay’). Tris Buffers, PBS/Phosphate Buffers and others
**Application as quoted in the referenced paper**	This briefly notes how the aptamer is used (e.g. Diagnostic, Therapeutic, Inhibition, Biosensor, Drug Delivery, Detection and Research)
**Pool Type**	Pool sequence, including the total nucleotide length of the random region nucleotides (ranging from 14 nt to 120 nt) and the number of random regions (e.g. 5′-contant_region-N40-contant_region-3′ or, in case of multiple random regions, 5′-constant_region-N5-constant_region-N9-constant_region-3′)
**Parent Sequence Serial Number**	This serial number refers to the original or parent aptamer sequence
**Post-SELEX modification**	Aptamer modification(s), if any, made after the initial aptamer selection/SELEX. Two common modifications are truncations and mutations
**Additional Information**	Inconsistencies, additional details about SELEX modification, if the pool was found in another publication, etc. are noted here
**Corresponding Author**	Last name and initial of first and middle name. If the corresponding author was not specified, the last author was noted as the corresponding author

**Table 2.** Summary of modified nucleic acids found in UTexas Aptamer Database aptamers

Nucleic acid modifications	
Modification (# of aptamers with modification)	Description
**2**′**-fluoro-RNA (80)**	Both pyrimidine nucleotides contain 2′-fluoro modifications (i.e. 2′-F-modified CTP and UTP & 2′-OH ATP and GTP)
**2**′**-amino-RNA (22)**	Includes 2 different types of modifications on one or both pyrimidine nucleotides: • Single CTP 2′-amino-modification (2′-NH_2_-CTP, 2′-OH-purine ATP/GTP/UTP) • Both pyrimidines (CTP and UTP) amino-modified (2′-NH_2_-pyrimidine, 2′-OH-purine RNA)
**2**′**-fluoro and 2**′**-amino-RNA (1)**	Both pyrimidine nucleotides have 2 different modifications (i.e. 2′-F-CTP, 2′-NH_2_, -UTP and 2′-OH ATP and GTP)
**2**′**-O-Me-RNA (5)**	Both pyrimidine nucleotides have 2′-O-Me modifications (i.e. 2′-O-methyl CTP and UTP and 2′-OH ATP and GTP)
**4**′**-thio-RNA (2)**	Both pyrimidine nucleotides have 4′-thio modifications instead of the 4′-O in the ribose ring (i.e. 4′-thio CTP and UTP, canonical ATP and GTP)
**5-uracil-modified-DNA (6)**	Modifications include 5-pentynyl-dU (5-(1-pentynyl)-2′-deoxyuridine) and Nap-dU (5-[*N*-(1-naphthylmethyl) Carboxamide]-2′-deoxyuridine)
**5-uracil-modified-RNA (1)**	Modification includes NH_2_-UTP (5-(3-aminopropyl) UTP)
**2**′**,4**′**-BNA/LNA-DNA (5)**	2′-O,4′-C-methylene bridged/locked nucleic acid
**FANA XNA (3)**	2′-deoxy-2′-fluoroarabinonucleotide (FANA) has unnatural structures (termed xeno-nucleic acids or XNA), containing 2′-fluorine in β-conformation and ring in the C2’/O4’-endo conformation (not typical 2′-fluoro α-conformation, ring in C3’-endo conformation)

For long-term preservation, easy maintenance and update, and multi-user accessibility of aptamer information, Google Sheets was selected to house the dataset. Ubiquitous in cloud services, Google Sheets has a history of stability and longevity as a web-based spreadsheet program, which integrates well with multiple programs and tools and provides a basal level of readability in its raw form. Versions of the dataset were routinely downloaded and backed up to ensure the information was preserved. The dataset includes aptamer information from the earliest paper published in 1990–2022 publications, consisting of complete, internally reviewed aptamer records. The information provided in Table 1 is a compilation of information collected from the paper for each aptamer record. In part, these categories were selected based on the necessary components for experimental reproducibility and previous and current aptamer database repositories (6,10). Additionally, each unique aptamer sequence was assigned an 8-digit serial number for cataloging and identification purposes, which provides an opportunity to cite and track individual sequence use cases, and modifications, and potentially trace the use through the literature.

The UTexas Aptamer dataset was also uploaded to the data repository Zenodo, which provides a unique DOI. This provides a snapshot of the data at the given time, allows another way for others to access the data, as well as meets the data deposition standards set by Nucleic Acids Research.

The data collection process was designed to adhere to a consistent data format, to report the aptamer sequences and to maintain the collection of sequences over time. In detail, most, if not all, aptamer papers from the first decade of aptamer research (1990–1999) were indexed in PubMed and included in the dataset. However, from 2000 to 2022, because of the many aptamer papers, we opted to include at least 15 papers per year.

PubMed was chosen as the primary search engine to identify aptamer literature, because a majority of the papers indexed on the site are primary research articles, and it provides few duplicate search results (i.e. similar rationale to the Dunn *et al.* 2017 work (11)). In PubMed, we used the search terms ‘aptamer’ and ‘aptamer SELEX’ and then filtered by ‘result by year’ (see Pubmed.gov slider on the left). In general, the first 10–30 papers for each of the given years were reviewed, and we attempted to extract sequence information. Extracted aptamer data were added to the dataset, while papers omitting aptamer sequences were not included in the dataset. Over time, aptamer data collectors began pulling papers with the additional term ‘Select’ in the title.

**Figure 2.**
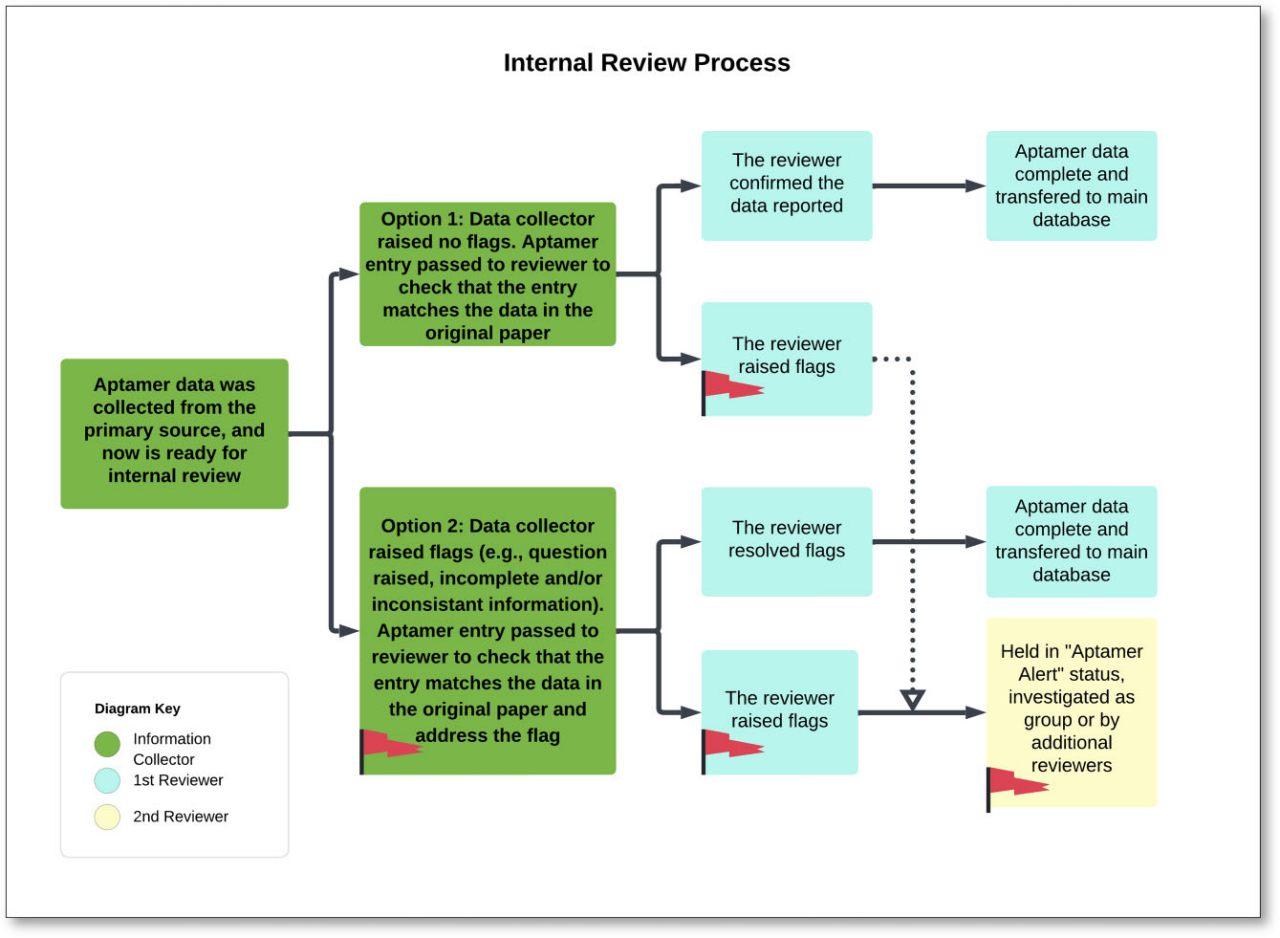
Flow chart displaying the internal review process of the aptamer sequence. The identity of the data collectors and the reviewers is logged.

Similar to the data collection methodology, we developed an internal review process. Briefly, internal reviewers examined the collected aptamer sequence data and reviewed it for accuracy after scanning the primary source, as described in the Figure 2 flow chart. To aid our process development, reviewers’ initials were recorded in the raw dataset, although not included in the database. Further, the information collection was standardized through the use of training videos, biweekly meetings and frequent consultation with established aptamer researchers.

**Figure 3.**
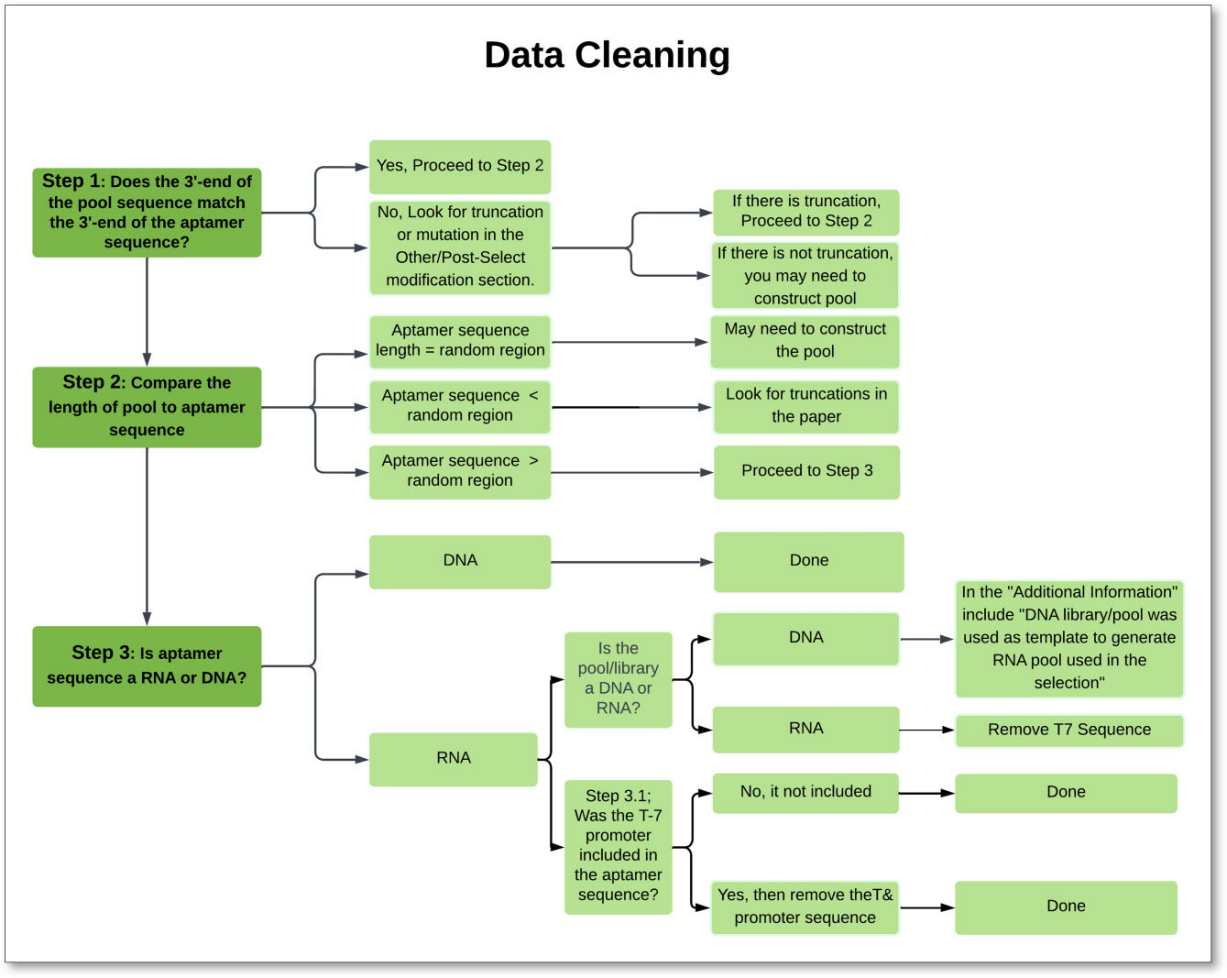
Data collectors and the internal reviewers used data cleaning throughout the process of building the dataset. Data cleaning, for example, checked the format of the data, that the pool could plausibly match the aptamer sequence, that the T7 promoter sequence was removed from RNA aptamers, etc.

After the internal review, an aptamer entry was deemed complete when most of the database fields/columns were filled and confirmed by at least one reviewer, while aptamer data with inconsistencies or collector/reviewer questions were further scrutinized. These scrutinized entries were internally described as having a ‘raised flag’. Some commonly raised flags included such cases as the selection buffer not being explicitly stated, the constant region in the pool did not match some of the aptamer sequences, the inability to construct the pool from the primers, or the pool being omitted entirely. Raised flags were discussed at team meetings, and if still unresolved, then the aptamer data were indefinitely moved to an ‘Aptamer Alert’ file for later review, without incorporation into the dataset or database until questions were resolved. Additionally, through all parts of the data collection and review process, data were regularly cleaned to properly format the data, fix common errors and check for consistency across fields for a given record (e.g. does the pool length match the length of the aptamer?) (see Figure 3).

**Figure 4.**
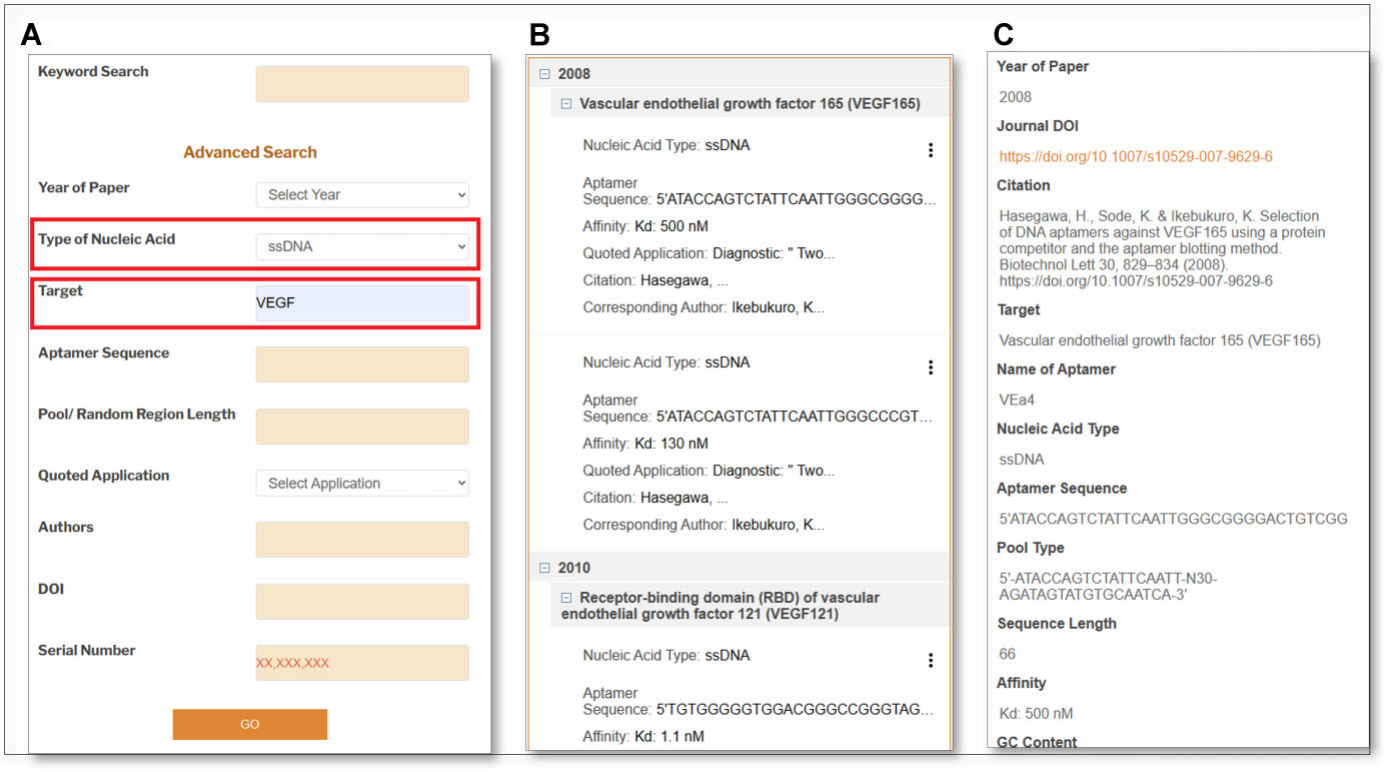
UTexas Aptamer Database screenshots and progression from Search to Details page: (A) Search page of the database, (b) the Results page provides a glance of some of the information collected for each aptamer, while clicking ‘View Details’ opens the Details page. (c) The Details page includes all the information about each aptamer record and a feedback system that users can use to leave comments about the accuracy of information.

The aptamer dataset is searchable via the Caspio online database platform, which is accessed through the host website in The University of Texas domain, https://sites.utexas.edu/aptamerdatabase/. This platform allows users to search by year of paper publication, type of nucleic acid, target, aptamer sequence, application referenced in the paper, pool/random region length, authors, DOI, serial number and keyword. Each search results page holds the aptamer sequence summaries (i.e. year of publication, target, type of nucleic acid, aptamer sequence, affinity, application as quoted in the referenced paper, citation and corresponding author category) and the page can be expanded to include additional details.

## Results and discussion

To develop the UTexas Aptamer Database (https://sites.utexas.edu/aptamerdatabase/), we extracted data from the literature for every year since the inception of aptamer selections, and included most of the aptamer sequences from a given paper (as opposed to just sequences with the tightest binding). This resulted in a collection of over 1400 aptamer sequences that spans decades (1990–2022). Since our dataset includes multiple sequences that emerged from a given selection experiment, it of necessity includes sequences that may not have been individually tested for binding activity, similar to the inclusion of all rRNA sequences in a metagenomic analysis of an environmental sample. By taking this metagenomic approach, we provide informaticians with a much wider range of sequences for subsequent analysis, while still providing tools to find high affinity aptamers for future use.

For each aptamer sequence, the database includes information about the aptamer publication (i.e. year of publication, DOI, full citation and corresponding author(s)), the aptamer target, as well as the following information about the specific aptamer: nucleic acid composition, name assigned in the original publication, sequence, GC percentage, sequence length, binding affinity (*K*_d_), binding/selection buffer, application as quoted in the referenced paper (e.g. drug delivery, biosensor, etc.), original nucleic acid pool used in the aptamer selection, post-selection modifications (if any), additional information and our internally assigned serial number (see Table 1 for additional descriptions of data collected). For each aptamer record, we used simple Excel formulas to calculate the GC content and the length of each aptamer sequence (see Supplementary Data Table S2). While all of the aptamer sequences have been reviewed for accuracy (see also Data curation, below), the homepage includes a disclaimer that reported aptamers have not undergone independent experimental validations for entry into the database. See Supplementary Data Figure S1 for screenshots of the raw data set.

In order to ensure long-term preservation and access, the data underlying this article are available in the UTexas Aptamer Database at https://sites.utexas.edu/aptamerdatabase/ and Zenodo at https://zenodo.org, and can be accessed with the DOI 10.5281/zenodo.8264921 or directly at https://zenodo.org/record/8264921. Notably, Zenodo provides best-in-class security and availability via CERN’s EOS distributed disk storage system.

### Search capabilities

The UTexas Aptamer Database uses the Caspio database platform, which is compatible with Chrome and Microsoft Edge browsers. The Database provides two search functionalities: Keyword Search and Advanced Search. The Advanced Search can provide users the ability to specify their search criteria via nine distinct fields (i.e. Year of Paper, Type of Nucleic Acid, Target, Aptamer Sequence, Possible Application, Pool/Random Region Length, Authors, DOI and Serial Number). The Database permits single search criterion (e.g. Year of Paper) or multiple search criteria (e.g. Year of Paper AND Target) to quickly narrow in on specific aptamers. In contrast, the Keyword Search function matches search terms across the various fields within the database, and thus may produce broader search results that encompass a wider range of relevant data.

Following a search, the Results page displays eight key details about the aptamer sequence. As an example, ssDNA aptamers against VEGF (vascular endothelial growth factor) can be found by selecting *ssDNA* in the ***Type of Nucleic Acid***drop-down window and typing *VEGF* into the ***Target*** search field (see Figure 4). For this search, the Results page revealed 6 cited aptamers:

- VEGF-165: 2 aptamers (Hasegawa, H., *et al.*, 2008),
- Receptor-Binding Domain (RBD) of VEGF165: 2 aptamers (Nonaka, Y, *et al.*, 2010) and
- RBD of VEGF121: 2 aptamers (Nonaka, Y, *et al.*, 2010).

Additional information on the Results page includes abbreviated information for each of the six aptamer records: Nucleic Acid Type, Aptamer Sequence (i.e. first 70 nt of the sequence; a complete view of the aptamer entry can be accessed by navigating to the ‘Details’ page), Affinity/*K*_d_, Citation (9 characters displayed) and Corresponding Author (12 characters displayed).

If more information is desired, users can click ‘View Details.’ For the first record, the anti-VEGF-165 aptamer with a *K*_d_ of 500 nM, the following information would appear:

- Year of Paper: 2008
- Journal DOI: https://doi.org/10.1007/s10529-007-9629-6
- Citation: Hasegawa, H., Sode, K. & Ikebukuro, K. Selection of DNA aptamers against VEGF165 using a protein competitor and the aptamer blotting method. Biotechnol Lett 30, 829–834 (2008). https://doi.org/10.1007/s10529-007-9629-6
- Target: Vascular endothelial growth factor 165 (VEGF165)
- Name of Aptamer: VEa4
- Nucleic Acid Type: ssDNA
- Aptamer sequence: 5′ATACCAGTCTATTCAATTGGGCGGGGACTGTCGGGATGTGTGTGGGCCAGATAGTATGTGCAATCA3′
- Pool type: 5′-ATACCAGTCTATTCAATT-N30-AGATAGTATGTGCAATCA-3′
- Sequence length: 68
- Affinity: *K*_d_: 500 nM
- GC content: 50%
- Binding buffer/conditions: TBSE (10 mM Tris/HCl, pH 7.0, 100 mM NaCl, 0.05 mM EDTA)
- Application as quoted in the referenced paper: Diagnostic: ‘Two DNA aptamers against a tumor marker protein, human vascular endothelial growth factor (VEGF(165)) were identified. This selection method enabled us to efficiently select the specific aptamers against the target protein. These specific aptamers would be useful sensor elements for cancer diagnosis’.
- Post-SELEX Modifications to the Aptamer: Not applicable.
- Additional Information: ssDNA library was labeled with FITC at the 5′ end.
- Corresponding Author Name, Email Address: Ikebukuro, K, ikebu@cc.tuat.ac.jp
- Serial number: 10,000,711
- Feedback form: https://forms.gle/n4TzuyddXQrHYJXF9

Each aptamer record includes a unique identifier (i.e. a serial number). Since aptamer nomenclature is not standardized, we have sometimes been challenged to align aptamer names with sequences. For example, we found that on some occasions a truncated aptamer was given the same name as the full-length aptamer, or that the name of an aptamer changed between publications. To provide standardization, we created a unique identifier in the form of an 8-digit number that appends to a particular aptamer sequence.

### Data curation

A previous effort from our research group had demonstrated that there may be a high error rate for accurately reporting aptamer sequences (2). While this issue can only be addressed as a community by the establishment of standards in databases and publications, we attempted to monitor our own efforts at standardization and curation by incorporating and comparing aptamer sequence results from another aptamer database: Apta-Index (Aptagen company in Jacobus, PA, https://www.aptagen.com/apta-index/). Apta-Index was originally chosen for two reasons: first, it is active as sequences are regularly added to the site; and second, it is sufficiently large for comparative purposes, containing almost 800 aptamer sequences (July 2023). There were 90 aptamers in common between the Apta-Index and our UTexas Aptamer Database. Of the 90 aptamers shared between databases, 27 differed between the datasets. Upon review, 14 were incorrectly entered in our database, and 13 were incorrectly entered in the Apta-Index (Supplementary Data Table S3). These discrepancies again indicate that one of the primary issues with aptamer research is the incorrect propagation of the sequences themselves. While we were able to discern that in this instance based upon database comparison, it obviously remains an issue for the future, for any database. In our instance, we are currently undertaking semi-annual reviews of a randomly selected set of entries, in order to better ensure accuracy into the future. We can provide some assurance to users that such reviews will continue into the future, as the UTexas Aptamer Database is a part of The University of Texas Freshman Research Initiative, a long-running (since 2005) and fully funded program (12–14, https://cns.utexas.edu/news/announcements/freshman-research-initiative-receives-higher-education-awards).

### Community participation

Into the longer term, the only way to ensure the accuracy and reproducibility of the aptamer literature is to encourage community participation in its archiving. There is no large-scale government-sponsored database for the collection and curation of aptamer sequences, such as the databases associated with EMBL (15), GeneBank (16) or DDBJ (17). Thus, one of the goals of the UTexas Aptamer Database is to make community participation possible (based on the search features we have implemented) and ultimately desirable (in terms of attracting other users to those search features). To this end, each record links to a feedback form, which provides users an opportunity to comment on the data within the record, note any results in attempting to reproduce work with the aptamer, or any other comments. This form is monitored by our UTexas Aptamer Database team, and the collected information is used to improve the quality of the data, and the user experience, and serves to provide the community with a means of ensuring self-validation of accuracy, building trust.

The UTexas Aptamer Database website also includes additional information and support pages for the community, in particular *Tips and Resources* pages and a *Submit an Aptamer* form. The *Tips and Resources* pages briefly describe the process for collecting aptamer sequences, our internal review criteria, descriptions of the nucleic acid modifications found in the database and contains a list of current and former aptamer databases (see Supplementary Data Table S1). The *Submit an Aptamer* form provides users an opportunity to submit aptamer information from a peer-reviewed publication. The information obtained from this form that meets our database standards (i.e. documented in peer-review publication) will be formatted for our database and incorporated into it. By providing standards and the opportunity for submission, we hope to begin the long process of self-curation by the community.

### Potential uses of the database

The UTexas Aptamer Database should be an extremely valuable resource for users. Previous efforts at publishing databases are relatively old (the Aptamer Database and Aptamer Base (6,10)). Aptamer Base appears to include only the top one or few aptamer sequences, those with the lowest *K*_d_ values, while our dataset includes most, or all, of the aptamer sequences provided in a given publication.

The UTexas Aptamer Database should be a resource for not only finding aptamers, but can be used by researchers to more generally determine the features of selected nucleic acids. For example, we analyzed the following: number of aptamer sequences/publications collected per year, count of aptamer publications collected by journal, aptamer nucleic acid composition (RNA vs. DNA), count of aptamers containing modified nucleotides, aptamer affinity versus C% (and versus G%), aptamer affinity versus nucleic acid type (RNA, DNA or modified nucleotides), aptamer affinity versus sequence length, aptamer affinity versus length of pool random region, aptamer affinity versus type of divalent salt, and aptamer affinity versus buffer type (Tris, PBS, or other). See Supplementary Data Figures S3–S4, and S6–S19. Based on these analyses, we find a slight skewing towards G and C in selected aptamers: RNA aptamers contained on average 30% G (SD 6.4%) and 24%C (SD 5.8%), and DNA aptamers contained on average 29% G (SD 8.4%) and 24% C (SD 6.2%) (see Supplementary Figure S5). However, when we examined the impact of aptamer GC content (GC%) on binding affinity; no significant correlation with aptamer binding affinity was observed (similar to previous analyses from McKeague *et al.* (10)). We also undertook to analyze the impact of buffer composition on aptamer selection and found no significant correlation (*R*^2^-value of 0.0059, see Supplementary Data Figure S2), a result that differed from McKeague *et al.*, who noted a weak correlation between higher pH buffers and improved binding affinity (10).

Importantly, we recognize that with the rise of machine learning tools that it may be possible to go beyond databases entirely, and just scry the open literature for answers. However, given the capabilities of such tools to ‘hallucinate’, the presence of a curated dataset is likely a much better starting point for acquiring and creating knowledge. To this end, we have also included a brief descriptor in the database regarding authors’ opinions regarding their own aptamers (‘Application as quoted in the referenced paper’). While we cannot vouch for the accuracy of the authors’ opinions, they will nonetheless provide a sourced, aggregated composite of words that are directly tied to known aptamers and should thereby prove extremely useful for interfacing the UTexas Aptamer DB with Large Language Models, such as ChatGPT.

### Conclusion

The UTexas Aptamer Database offers a broad and increasing sampling of aptamer sequences and selection data across decades, and now includes over 1400 aptamer sequences from more than 480 papers. It includes search tools for a diversity of applications, including finding aptamers for a given target molecule, scanning buffer conditions for a new selection, and analyzing the characteristics of selected sequences as a whole. By providing a framework for curating disparate selection experiments and resultant sequences, we hope to encourage scrutiny and submissions from the broader community, which should ultimately result in better standards for carrying out selection experiments and reporting data. Into the future, ganging data across multiple different categories, including delimited portions of text from the papers themselves, should help provide datasets for training AI models.

## Supplementary Material

gkad959_supplemental_file

## Data Availability

The data underlying this article are available in the UTexas Aptamer Database at https://sites.utexas.edu/aptamerdatabase/ and Zenodo at https://zenodo.org, and can be accessed with the DOI 10.5281/zenodo.8264921 or directly at https://zenodo.org/record/8264921. Additional data underlying this article are available in the article and its online supplementary material.

## References

[1] DeRosa M.C. , Lin A., Mallikaratchy P., McConnell E.M., McKeague M., Patel R., Shigdar S. In vitro selection of aptamers and their applications. Nat. Rev. Methods Primers. 2023; 3:54. 10.1038/s43586-023-00247-6 PMC10647184 37969927

[2] Miller A.A. , Rao A.S., Nelakanti S.R., Kujalowicz C., Shi T., Rodriguez T., Ellington A.D., Stovall G.M. Systematic review of aptamer sequence reporting in the literature reveals widespread unexplained sequence alterations. Anal. Chem. 2022; 94:7731–7737. 35420426 10.1021/acs.analchem.1c04407 PMC9179646

[3] McKeague M. What happened to all the aptamer databases?. Int. Soc. Aptamers. 2021; VI:2.

[4] Wu Y.X. , Kwon Y.J. Aptamers: the “evolution” of SELEX. Methods. 2016; 106:21–28. 27109056 10.1016/j.ymeth.2016.04.020

[5] Cruz-Toledo J. , McKeague M., Zhang X., Giamberardino A., McConnell E., Francis T., DeRosa M.C., Dumontier M. Aptamer base: a collaborative knowledge base to describe aptamers and SELEX experiments. Database. 2012; 2012:bas006. 22434840 10.1093/database/bas006 PMC3308162

[6] Lee J.F. , Hesselberth J.R., Meyers L.A., Ellington A.D. Aptamer database. Nucleic Acids Res. 2004; 32:90001. 10.1093/nar/gkh094 PMC308828 14681367

[7] Ellington A.D. , Szostak J.W. In vitro selection of RNA molecules that bind specific ligands. Nature. 1990; 346:818–822. 1697402 10.1038/346818a0

[8] Tuerk C. , Gold L. Systematic evolution of ligands by exponential ENRICHMENT: RNA LIGANDS to bacteriophage T4 DNA polymerase. Science. 1990; 249:505–510. 2200121 10.1126/science.2200121

[9] Baird G.S. Where are all the Aptamers?. Am. J. Clin. Pathol. 2010; 134:529–531. 20855632 10.1309/AJCPFU4CG2WGJJKS

[10] McKeague M. , McConnell E.M., Cruz-Toledo J., Bernard E.D., Pach A., Mastronardi E., Zhang X., Beking M., Francis T., Giamberardino A. et al. Analysis of In Vitro aptamer selection parameters. J. Mol. Evol. 2015; 81:150–161. 26530075 10.1007/s00239-015-9708-6

[11] Dunn M. , Jimenez R., Chaput J. Analysis of aptamer discovery and technology. Nat Rev Chem. 2017; 1:0076.

[12] Rodenbusch S.E. , Hernandez P.R., Simmons S.L., Dolan E.L. Early engagement in course-based research increases graduation rates and completion of science, engineering, and mathematics degrees. CBE—Life Sci, Educ. 2016; 15:ar20. 27252296 10.1187/cbe.16-03-0117 PMC4909342

[13] Walcott R.L. , Corso P.S., Rodenbusch S.E., Dolan E.L. Benefit–cost analysis of undergraduate education programs: an example analysis of the Freshman Research Initiative. CBE—Life Sci. Educ. 2018; 17:rm1. 29378752 10.1187/cbe.17-06-0114 PMC6007785

[14] Stovall G.M. , Huynh V., Engelman S., Ellington A.D. Aptamers in education: undergraduates make aptamers and acquire 21st century skills along the way. Sensors. 2019; 19:3270. 31349595 10.3390/s19153270 PMC6696043

[15] Cochrane G. , Akhtar R., Bonfield J., Bower L., Demiralp F., Faruque N., Gibson R., Hoad G., Hubbard T., Hunter C. et al. Petabyte-scale innovations at the European Nucleotide Archive. Nucleic Acids Res. 2009; 37:D19–D25. 18978013 10.1093/nar/gkn765 PMC2686451

[16] Benson D.A. , Cavanaugh M., Clark K., Karsch-Mizrachi I., Lipman D.J., Ostell J., Sayers E.W. GenBank. Nucleic Acids Res. 2012; 41:D36–D42. 23193287 10.1093/nar/gks1195 PMC3531190

[17] Kaminuma E. , Mashima J., Kodama Y., Gojobori T., Ogasawara O., Okubo K., Takagi T., Nakamura Y DDBJ launches a new archive database with analytical tools for next-generation sequence data. Nucleic Acids Res. 2010; 38:D33–D38. 19850725 10.1093/nar/gkp847 PMC2808917

