# Supplementary material for: UTexas Aptamer Database: the collection and long-term preservation of aptamer sequence information
Source: Nucleic Acids Res. 2023 Oct 30;52(D1):D351–9. doi: 10.1093/nar/gkad959 (PMC10767891; doi:10.1093/nar/gkad959)
Supplement: gkad959_supplemental_file [file gkad959_supplemental_file.pdf]

Supplementary Data

UTexas Aptamer Database: The Collection and Long-Term Preservation of Aptamer Sequence Information

AUTHORS

Ali Askari[1], Sumedha Kota[1], Hailey Ferrell[1], Shriya Swamy[1], Kayla S. Goodman[1], Christine C. Okoro[1], Isaiah C. Spruell Crenshaw[1], Daniela K. Hernandez[1], Taylor E. Oliphant[1], Akshata A. Badrayani[1], Andrew D. Ellington[3,4], Gwendolyn M. Stovall[1,2,3]*

[1] Freshman Research Initiative, The University of Texas, Austin, Texas, 78712, USA

[2] High School Research Initiative, The University of Texas, Austin, Texas, 78712, USA

[3] Institute for Molecular Biosciences, The University of Texas, Austin, Texas, 78712, USA

[4] Center for Systems and Synthetic Biology, The University of Texas, Austin, Texas, 78712, USA

* To whom correspondence should be addressed. Tel: 512-471-4049; Fax: 512-232-1045; Email: gwenstovall@utexas.edu

Disclaimer:

The information provided in this analysis is based on data available as of July 2023 and may not reflect the most current data. Therefore, any decisions or actions taken based on this information should be carefully evaluated in light of more recent and relevant data.

Table of Content

Table S1: A summary of aptamer databases in order of launch date. 3

Table S2: Formulas used in the Google Sheets (e.g., calculate GC content, sequence length, etc.)..4

Figure S1: A screenshot of the UTexas Aptamer dataset in Google Sheets. 5

Figure S2: Aptamer binding affinity vs. pH. 6

Figure S3: Aptamer binding affinity in nM vs. GC%. 6

Figure S4: Aptamer binding affinity in nM vs. GC% (boxplot). 7

Figure S5: Frequency of each base per aptamer sequence. 7

Figure S6: Number of aptamer sequences and aptamer publications per year.........................8

Figure S7: Count of aptamer publications by journals, according to the UTexas Aptamer dataset. 8

Figure S8: Summary of the nucleic acid type of aptamer sequence found in the UTexas Aptamer Database. 9

Figure S9: Count of each nucleic acid type in the UTexas Aptamer Database. 9

Figure S10: Aptamer binding affinity vs. aptamer C%-base count. 10

Figure S11: Aptamer binding affinity in nM vs. C% (boxplot). 10

Figure S12: Aptamer binding affinity vs G%-Base count in the aptamer sequence. 11

Figure S13: Aptamer binding affinity in nM vs. G% (boxplot). 11

Figure S14: Nucleic acid type versus aptamer affinity (boxplot).......................................12

Figure S15: Aptamer binding affinity versus sequence length. 12

Figure S16: Aptamer binding affinity versus pool random region......................................13

Figure S17: Length of pool random region versus aptamer affinity (boxplot). 13

Figure S18: Divalent salt included in binding buffer versus aptamer affinity (boxplot)...............14

Figure S19: Buffer type versus aptamer binding affinity (boxplot). 14

Table S3: Comparison of Aptamer Databases: Examination of those sequences that differed..21

Database Name	Institution	Aptamer Sequences	Current Status	First launched or date of publication	Reference
SELEX_DB	Institute of Cytology and Genetics	116	Last updated 2015	2000	Ponomarenko, et al., 2000 & 2001
Aptamer Database*	The University of Texas at Austin	239	Inactive	2004	Lee, et al., 2004
RiboaptDB*	University of Southern Mississippi	3842	Inactive	2006	Thodima, et al., 2006
HTPSELEX	Swiss Institute for Experimental Cancer Research and Swiss Institute of Bioinformatics	Not determined	Last updated September 2012	2006	Jagannathan, et al., 2006
Apta-Index™	Aptagen	783 (as of April 2023)	Active and updated	2008	No paper to date.
Aptamer Base*	Carleton University	2334	Inactive (CSV file with aptamer sequence information is available on GitHub)	2012	Cruz-Toledo, et al., 2012
Aptabase	Indian Institute of Technology Guwahati	605	Periodically updated	2021	No paper to date.

Table S1: A summary of aptamer databases in order of launch date.

*Indicates the database management included mechanisms to check the accuracy of the sequences (e.g., “Aptamer Base” relied on users' wiki-contributions to correct or augment the dataset).

Function	Formula	Field used	Conditional formatting or Cell
Finding duplicates	=countif(D:D,D1)>1	Link to PubMed Entry (green), Journal DOI (light orange), Citation (Light blur), Aptamer sequence (yellow)	Conditional formatting
Finding duplicate in across multiple sheets/column.	Info Collect-setup<br>=match($E1:$E, indirect("Database!D2:D"),0)<br><br>Database Sheet<br>=match($E2:$E, indirect("Database!D2:D"),0)	DOI. This was used to see if the doi in Info collect-setup sheet is the same as main database.	Conditional formatting
Sequence Length	=(LEN(I2)- LEN(SUBSTITUTE(I2,"G","")))+ (LEN(I2)-LEN(SUBSTITUTE(I2,"C",""))) +(LEN(I2)-LEN(SUBSTITUTE(I2,"A",""))) +(LEN(I2)-LEN(SUBSTITUTE(I2,"T","")))+ (LEN(I2)-LEN(SUBSTITUTE(I2,"U",""))) + (LEN(I2)- LEN(SUBSTITUTE(I2,"g","")))+ (LEN(I2)-LEN(SUBSTITUTE(I2,"c",""))) +(LEN(I2)-LEN(SUBSTITUTE(I2,"a",""))) +(LEN(I2)-LEN(SUBSTITUTE(I2,"t","")))+ (LEN(I2)-LEN(SUBSTITUTE(I2,"u","")))	Sequence length	Cell
GC content calculation	=((LEN(I35)- LEN(SUBSTITUTE(I35,"G","")))+ (LEN(I35)- LEN(SUBSTITUTE(I35,"C","")))+ (LEN(I35)- LEN(SUBSTITUTE(I35,"g","")))+ (LEN(I35)- LEN(SUBSTITUTE(I35,"c",""))))/J35	GC content	Cell

Table S2: Formulas used in the Google Sheets (e.g., calculate GC content, sequence length, etc.).

a.

Name of Information Collectors [Initials of reviewers]	Year of Paper	Link to PubMed Entry	Journals	Journal DOI	Citation	Type of Nucleic Acid	Name of Aptamer	Target	Aptamer Sequence	Sequence Length	GC Content	Affinity	Kd (nM)	Pool Type	Pool Random Region	Binding Buffer/Conditions
Ali Askari [ISC]	2008	https://pubmed.r	J Agric Food Che	https://doi.org/1(	Cruz-Aguado,	ssDNA	1.12	Ochratoxin A (	5'TGGTGGCTGTAGGTCA	61	59%	Kd: 0.36	0.36	5'TGGTGGCTGTAG	30	Selection buffer (SB
Isaac Weislow [AA] [KG]	2000	https://pubmed.r	Bioorg Med Che	https://doi.org/1(	Fukusaki, E.,	ssDNA	Chi No 52	Poly-beta-1,4-	5'TAGGGAATTCGTCGA	105	60%	Binding Efficie	N/A	5'-TAGGGAATTCGT	59	100 mM NaCl, 100
Sumedha Kota [PA]	2000	https://pubmed.r	Bioorg Med Che	https://doi.org/1(	Okazawa, A.,	ssDNA	26	Hematoporphy	5'TAGGGAATTCGTCGA	102	67%	Kd: 1.6x10^-6	1600	5'-TAGGGAATTCGT	59	100 mM Tris±acetat
Sumedha Kota [KG]	1996	https://pubmed.r	J Clin Invest	https://doi.org/1(	Hicke, B. J., V	ssDNA	LD201t1	L-selectin–IgG	5'TAGCCAAGGTAACCAG	49	47%	Not reported	N/A	5'-CTACCTACGATC	40	20 mM Hepes, pH 7

b.

Pool Random Region	Binding Buffer/Conditions	Dividant Salt	Type of the buffer	pH	Molecular weight of target	Application as quoted in the referenced paper	Post-selex modifications to the aptamer	Additional Relevant Information	Serial Number	Corresponding Author Name, email address	please fill out the form for any feedbacks/comments	Aptagen Cross Referencing(Check Aptamer Chemistry, Affinity, Length, GC content, sequence)
30	Selection buffer (SB	MgCl/CaCl	Tris Buffers	7.0	Not reported	Research and Detection: "This work describes the identification of an aptamer that binds		The library wa	10,000,654	Penner, G, E-mail: g	https://forms.gle	5'dTpdGpdGpdT<br>https://www.apta
59	100 mM NaCl, 100	MgCl	Tris Buffers	8.0	Not reported	Diagnostic: " Oligosaccharide antigens play essential biological rol	Not applicable	G-cluster mot	10,000,317	Fukusaki E, fukusaki	https://forms.gle	5'dTpdApdGpd<br>Aptamer length<br>https://www.ap
59	100 mM Tris±acetat	None	Tris Buffers	8.0	Not reported	Detection: " In the present study, we selected single-stranded DNA	Not applicable		10,000,301	Kobayashi, A, kobay	https://forms.gle	5'dTpdApdGpd<br>Aptamer Lengt<br>https://www.ap
40	20 mM Hepes, pH 7	MgCl/CaCl	Other Buf...	7.5	Not reported	Therapeutic: " Aptamers that bind with nanomolar affinity to L-selectin's lectin domain, prevent L-selec			10,000,139	Parma, D, parma@n	https://forms.gle	5'dTpdApdGpd<br>Aptamer kd is c<br>https://www.ap
35	CE Buffer (30 mM N	None	PBS/pho...	7.5	Not reported	Diagnostic and Therapeutic: " Alpha-fetoprotein (AFP) is a liver car	Not applicable		10,001,063	Guo, W., guo.wei@z	https://forms.gl	5'dGpdTpdGpc<br>https://www.ap

Figure S1: A screenshot of the UTexas Aptamer dataset in Google Sheets.

The name of information collectors and initials of reviewers were recorded in the first column to track systematic errors across the database. Bright blue indicate there is more than one entry from one publication. Figure 1 B is the continuation of Figure 1 A.

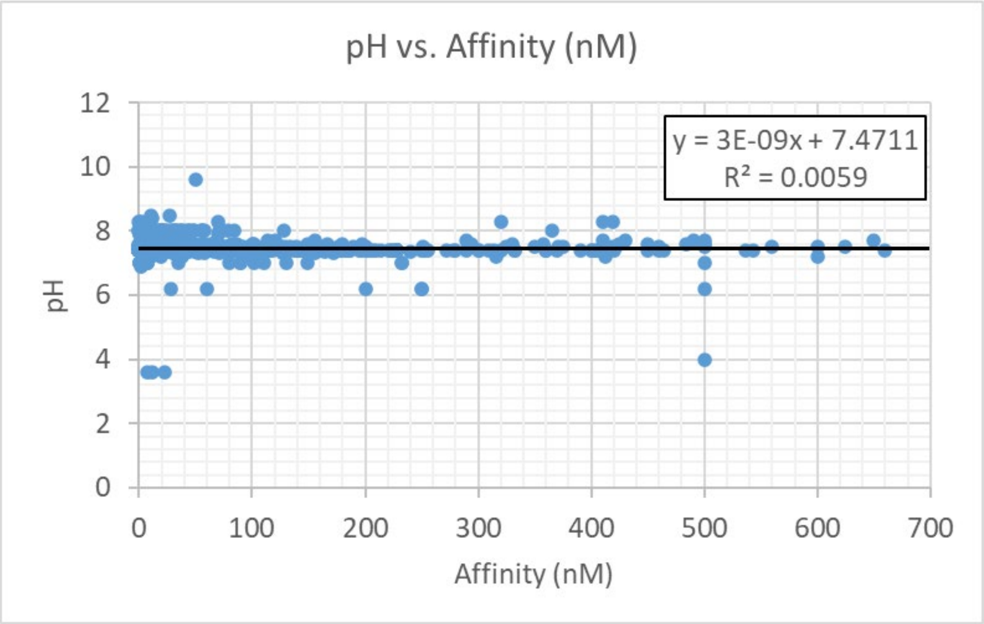

Figure S2: Aptamer binding affinity vs. pH.

The relationship between these two variables is assessed using the line of best fit. With an R^2 value of 0.0059, there is no correlation between the two variables. The y-axis was adjusted to a zoom level that selectively displays data up to 700 nM.

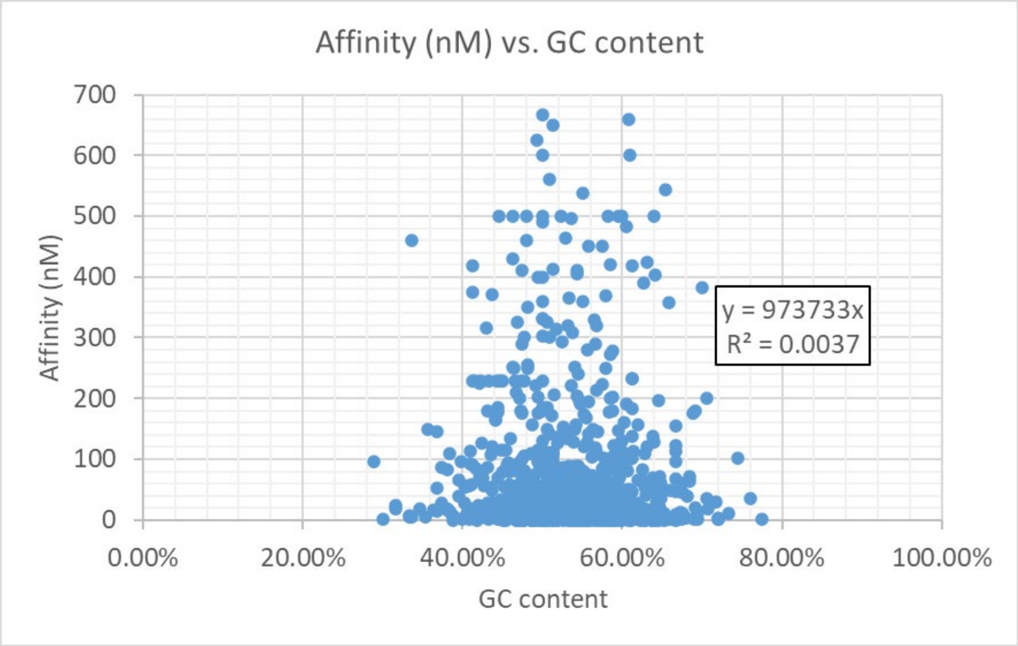

Figure S3: Aptamer binding affinity in nM vs. GC%.

The relationship between these two variables is assessed using the line of best fit. With an R^2 value of 0.0037, there is no correlation between the two variables. The y-axis was adjusted to a zoom level that selectively displays data up to 700 nM, and the intercept was set at zero.

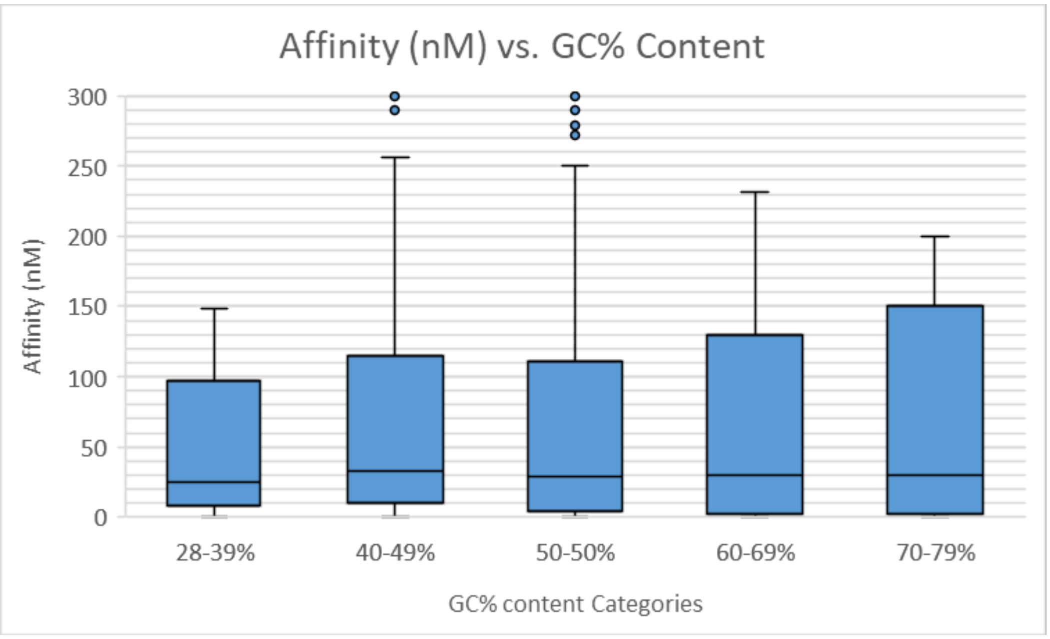

Figure S4: Aptamer binding affinity in nM vs. GC% (boxplot).

There are five GC% categories to assess whether the specific range of GC% affect affinity. The y-axis was adjusted to a zoom level that selectively displays data up to 300 nM.

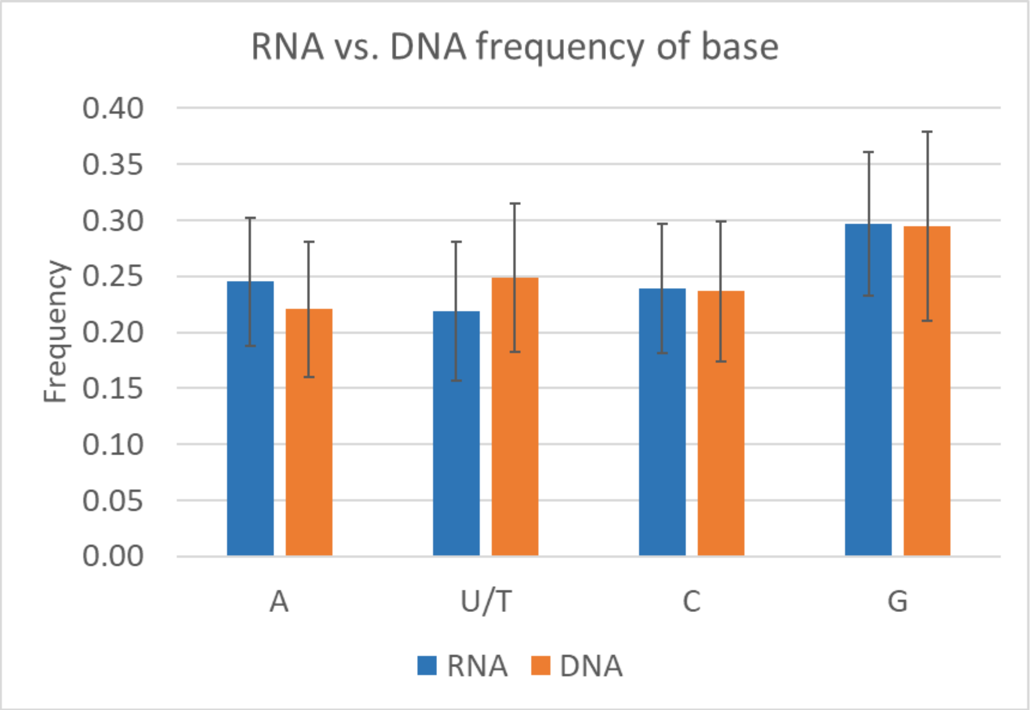

Figure S5: Frequency of each base per aptamer sequence.

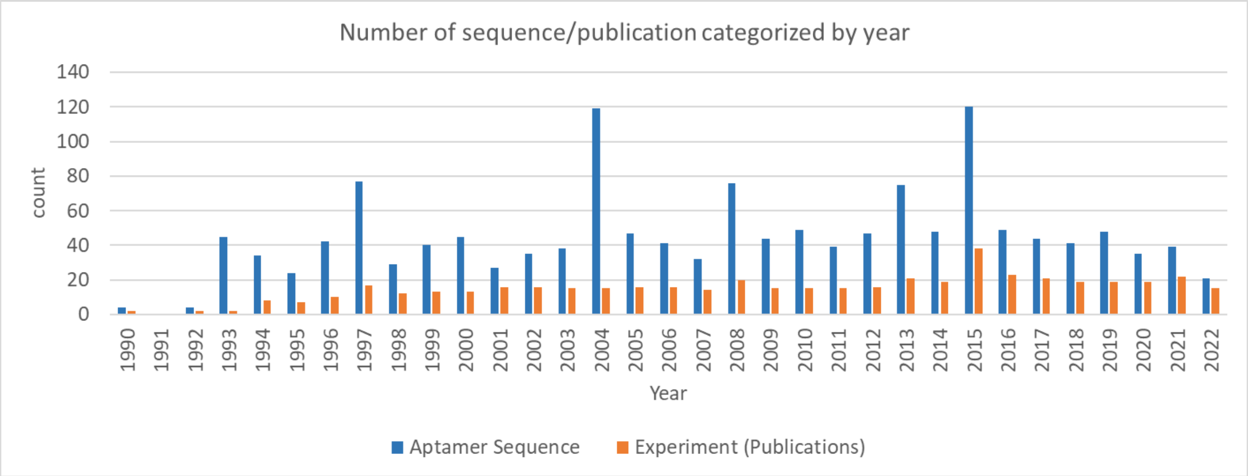

Figure S6: Number of aptamer sequences and aptamer publications per year.

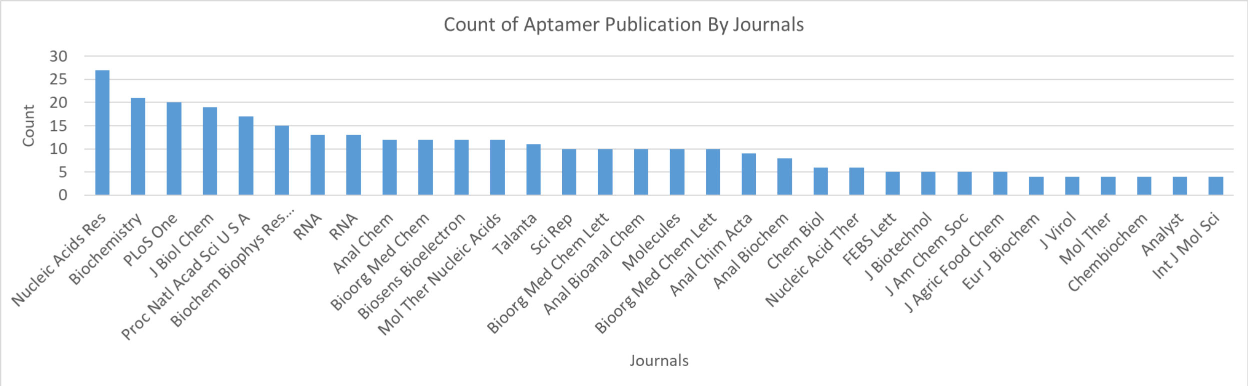

Figure S7: Count of aptamer publications by journals, according to the UTexas Aptamer dataset.

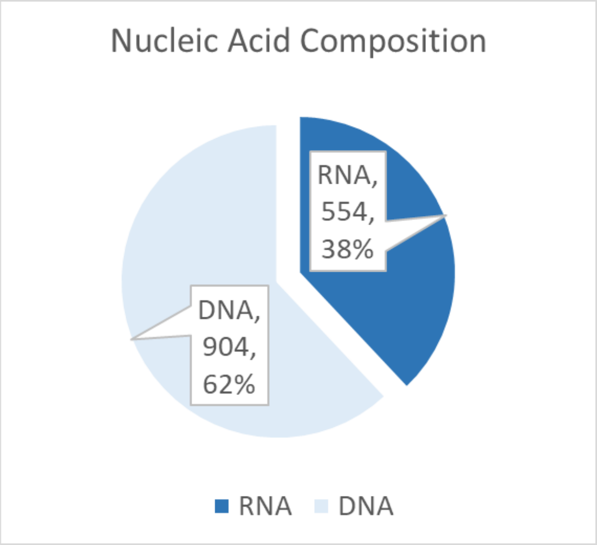

Figure S8: Summary of the nucleic acid type of aptamer sequence found in the UTexas Aptamer Database.

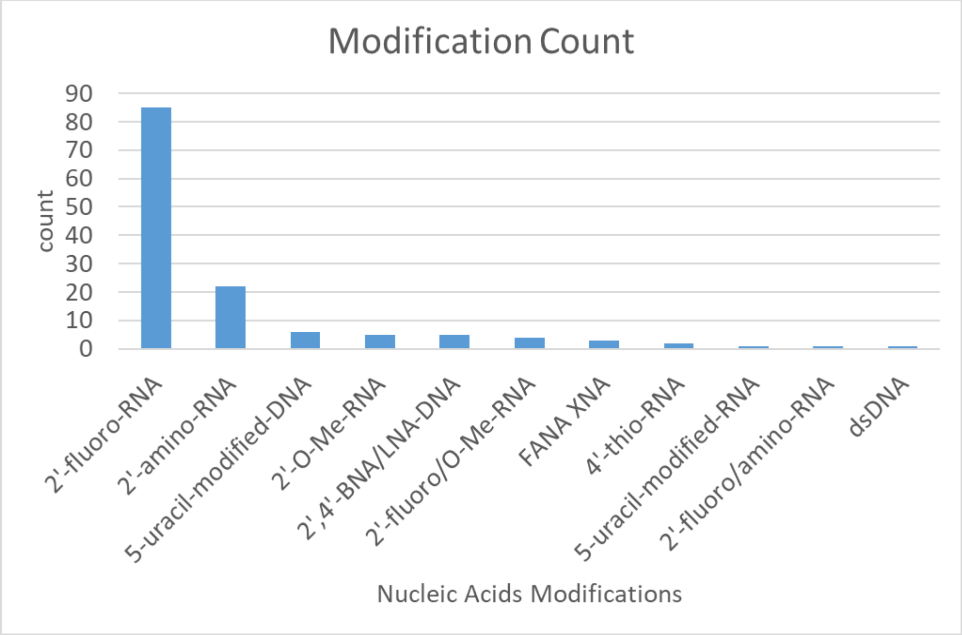

Figure S9: Count of each nucleic acid type in the UTexas Aptamer Database.

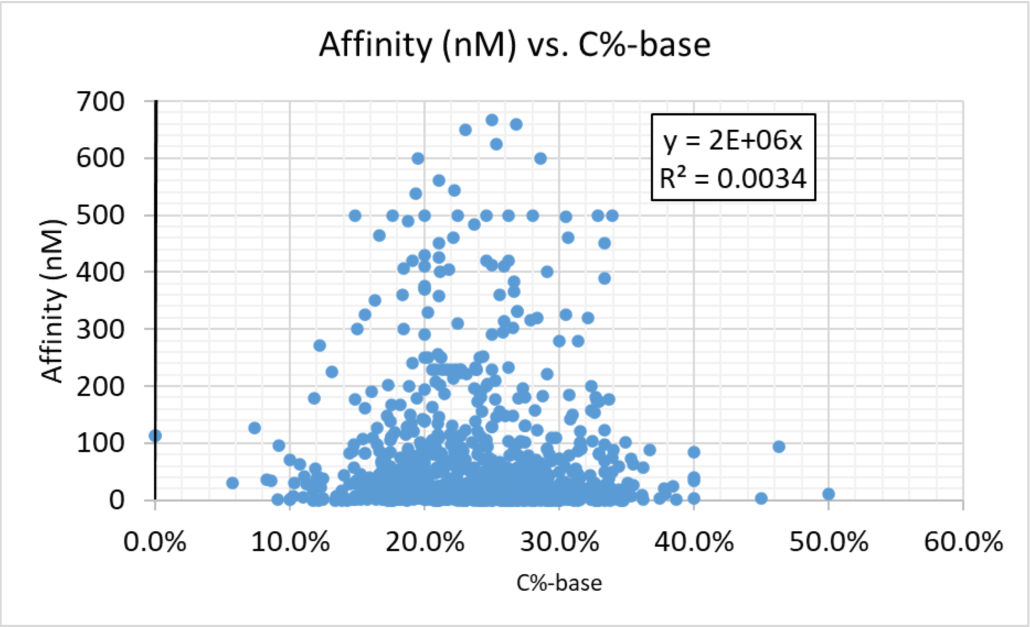

Figure S10: Aptamer binding affinity vs. aptamer C%-base count.

The relationship between these two variables is assessed using the line of best fit. With an R^2 value of 0.0034, there is no correlation between the two variables. The y-axis was adjusted to a zoom level that selectively displays data up to 700 nM, and the intercept was set at zero.

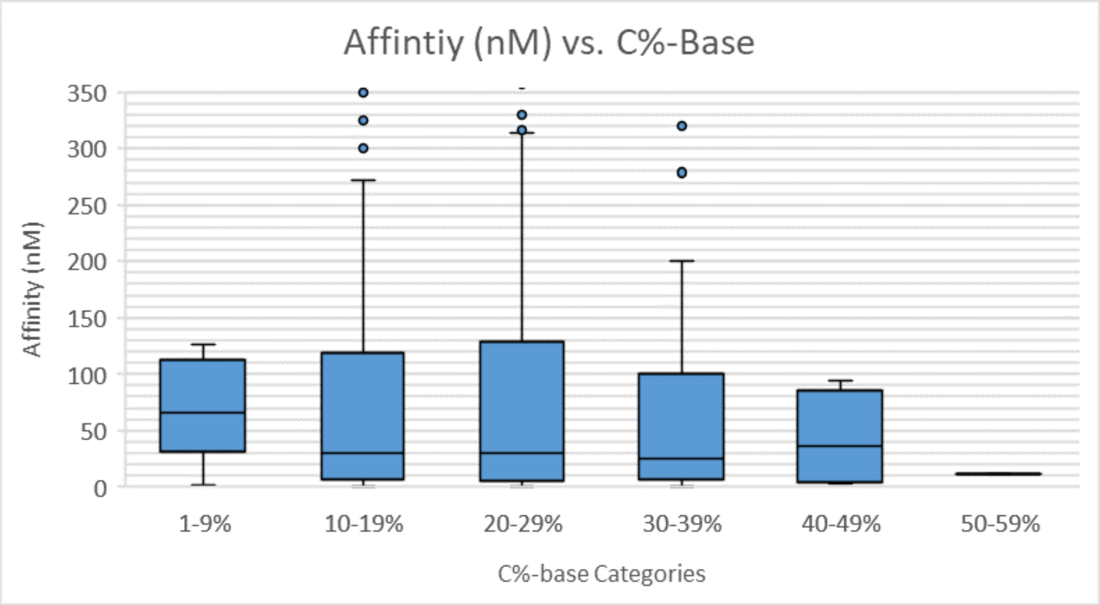

Figure S11: Aptamer binding affinity in nM vs. C% (boxplot).

There are six C% categories to asses whether the specific range of C% affect affinity. Categories 1-9%, 40-49%, and 50–59% have less than 20 sample sizes. The y-axis was adjusted to a zoom level that selectively displays data up to 350 nM.

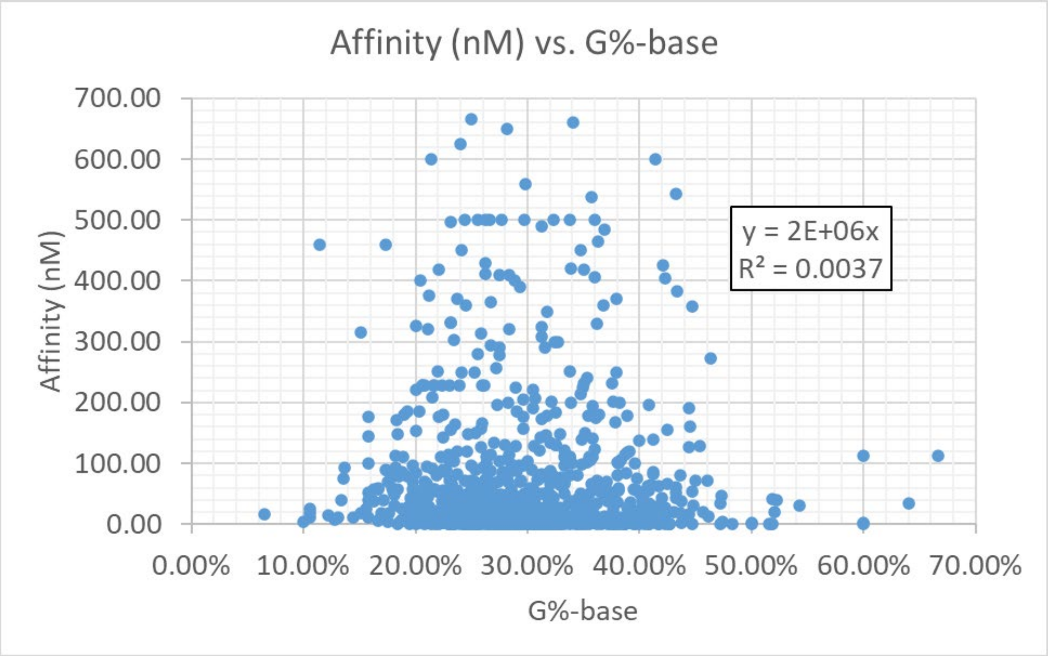

Figure S12: Aptamer binding affinity vs G%-Base count in the aptamer sequence.

The relationship between these two variables is assessed using the line of best fit. With an R^2 value of 0.0037, there is no correlation between the two variables. The y-axis was adjusted to a zoom level that selectively displays data up to 700 nM, and the intercept was set at zero.

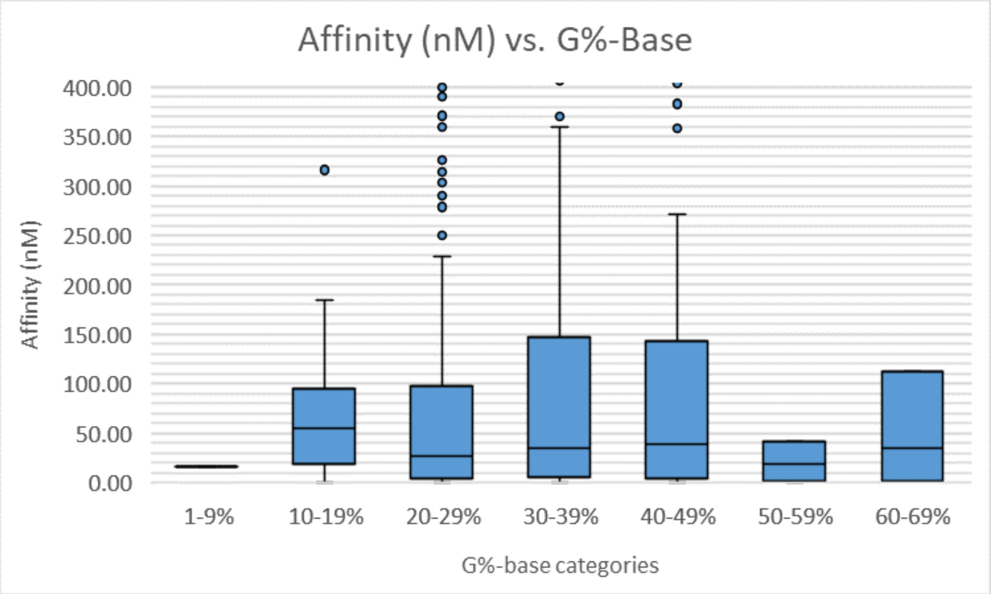

Figure S13: Aptamer binding affinity in nM vs. G% (boxplot).

There are six G% categories to assess whether the specific range of G% affects affinity. Categories 1-9%, 50–59%, and 60-69% have less than 20 sample sizes. The y-axis was adjusted to a zoom level that selectively displays data up to 400 nM.

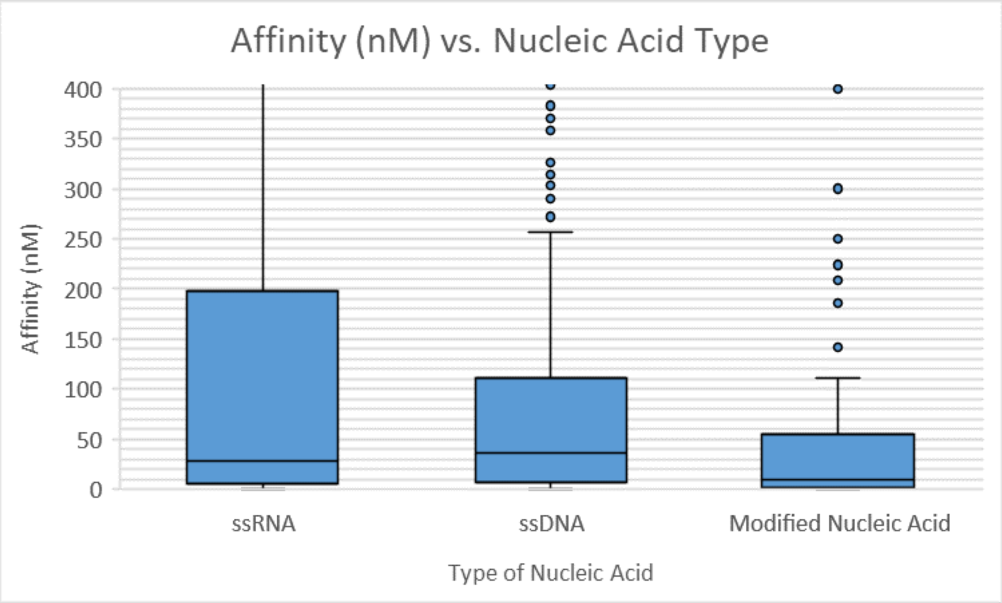

Figure S14: Nucleic acid type versus aptamer affinity (boxplot).

The y-axis was adjusted to a zoom level that selectively displays data up to 400 nM.

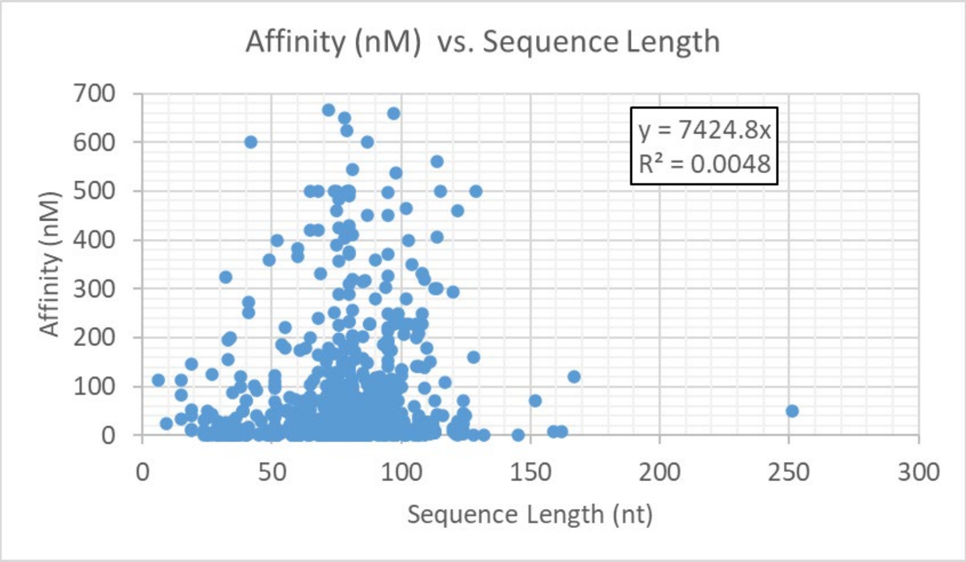

Figure S15: Aptamer binding affinity versus sequence length.

The relationship between these two variables is assessed using the line of best fit. With an R^2 value of 0.0048, there is no correlation between the two variables. The y-axis was adjusted to a zoom level that selectively displays data up to 700 nM, and the intercept was set at zero.

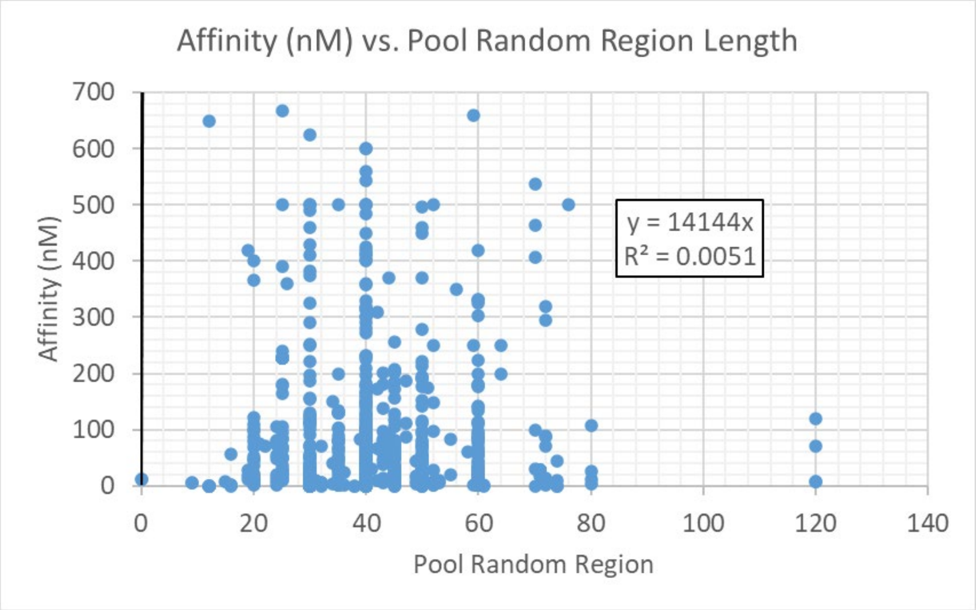

Figure S16: Aptamer binding affinity versus pool random region.

The relationship between these two variables is assessed using the line of best fit. With an R^2 value of 0.0051, there is no correlation between the two variables. The y-axis was adjusted to a zoom level that selectively displays data up to 700 nM, and the intercept was set at zero.

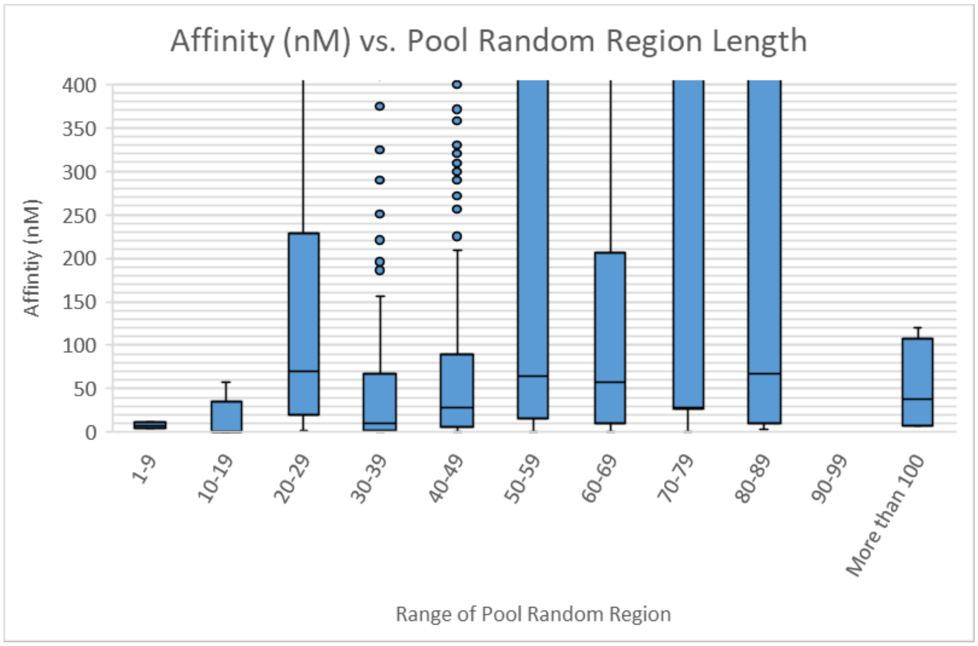

Figure S17: Length of pool random region versus aptamer affinity (boxplot).

Categories 1-9, 10-19, 80-89, 90-99, and more than 100 have less than 20 sample sizes. The y-axis was adjusted to zoom-in on the area around the boxplots, data up to 400 nM displayed.

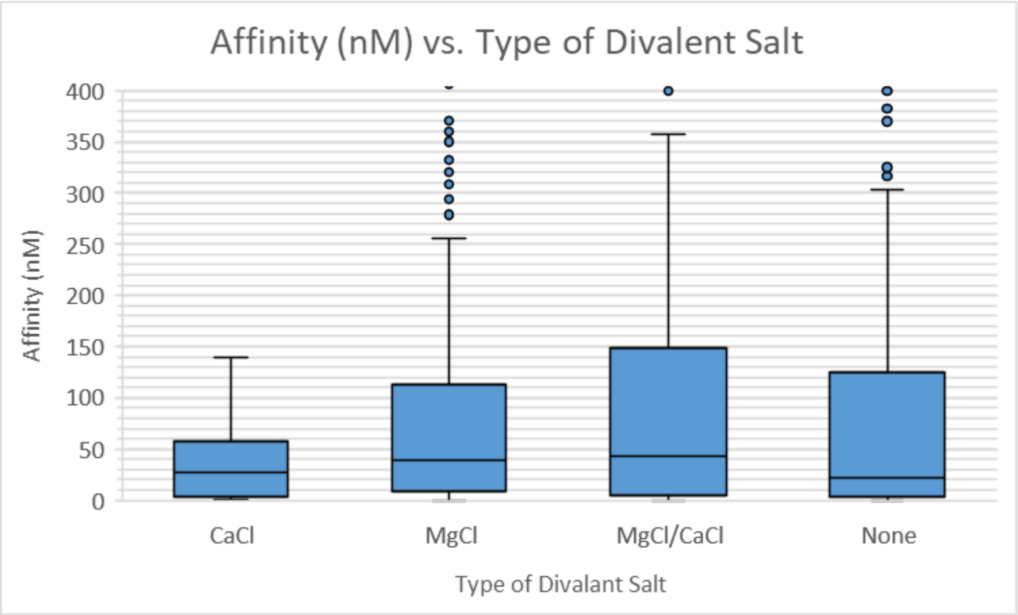

Figure S18: Divalent salt included in binding buffer versus aptamer affinity (boxplot).
The y-axis was adjusted to a zoom level that selectively displays data up to 400 nM.

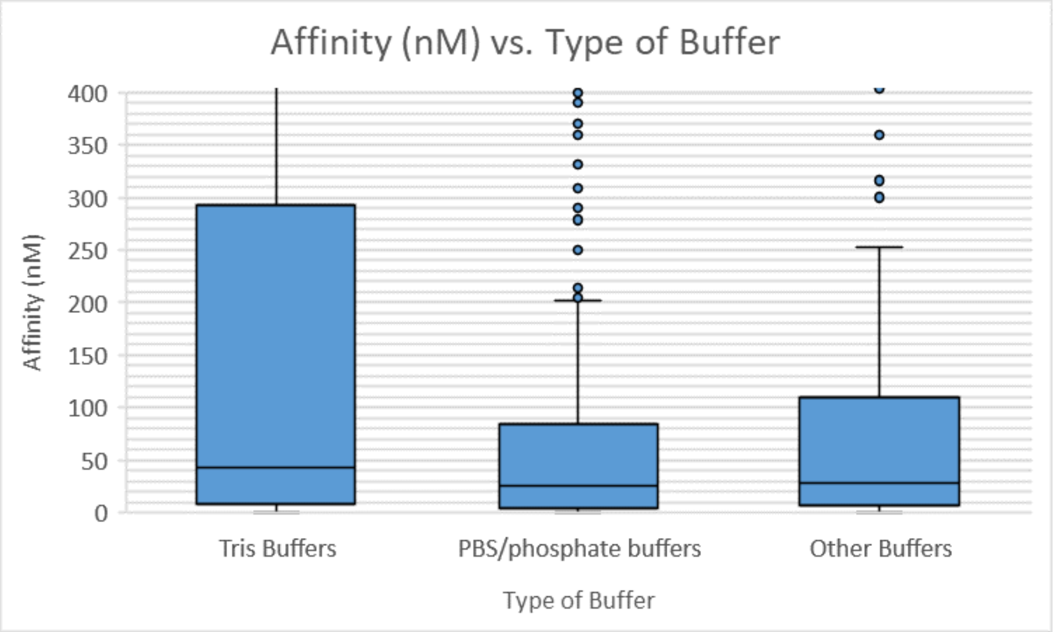

Figure S19: Buffer type versus aptamer binding affinity (boxplot).
The y-axis was adjusted to zoom-in on the area around the boxplot, data up to 400 nM displayed.

UTexas DB Serial Number and Citation	Aptamer Name, Target<br><br>Sequence Comparison	Source of Error
# 10000063<br><br>Lorsch, J. R., & Szostak, J. W. (1994). Biochemistry, 33(4), 973-982.	35-mer aptamer, anti-Cobinamide dicyanide<br><br>`UTexas:     5'GGAACCGGUGCGCAUAACCACCUCAGUGCGAGCAA3'`<br>`Apta-Index: 5'----CCGGUGCGCAUAACCACCUCAGUGCGAGCAAGGAA3`	Apta-Index added GGAA to wrong end
# 10001000<br><br>Kwon, H. M., et al. (2014). A PloS one, 9(5), e97574.	HA12-16, anti-gHA1<br><br>`UTexas:     5'GGGUUCACUGCAGACUUGACGAAGCUUGCUUGACGGAGAUCAAGGGCGAGUCGCAUACCAAGUUGAUGGGGAAUGGAUCCACAUCUACGAAUUC3'`<br>`Apta-Index: 5'GACAAGGATAAATCCTTCAATGAAGTGGGTCACTCATCTGTGA3'`	Apta-Index reported an entirely different sequence.
# 10001063<br><br>Dong, L., et al. (2015). Scientific reports, 5(1), 15552.	AP273, anti-Alpha-fetoprotein (AFP)<br><br>`UTexas:`<br>`5'GTGACGCTCCTAACGCTGACGTGACGCTCCTAACGCTGACTCAGGTGCAGTTCTCGACTCGGTCTTGATGTGGGTCCTGTCCGTCCGAACCAATCCCTGTCCGTCCGAACCAATC3'`<br>`Apta-Index:          5'GTGACGCTCCTAACGCTGACTCAGGTGCAGTTCTCGACTCGGTCTTGATGTGGGTC-TGTCCGTCCGAACCAATC3'`	Both had errors: UTexas duplicated primer regions and Apta-Index deleted an internal C.
#10001073<br><br>Li, P., et al. (2015). BMC Veterinary Research, 11(1), 1-11.	QA-36, anti-Soft-shelled turtle iridovirus (STIV)<br><br>`UTexas: 5'GACGCTTACTCAGGTGTGACTCGTGTGCGGGGGAGGGGAGTGGCGCTGTTGGTGCGGGTATAGCGCGTGGTGTCGAAGGACGCAGAGAAGTCTC3'`<br>`        5'-----------------------TGTGCGGGGGAGGGGAGTGGCGCTGTTGGTGCGGGTATAGCGCGTGGTGT---------------------3'`<br>`^ Apta-Index:`	Apta-Index deleted the primer regions.

# 10001268<br><br>Xiang, J., et al. (2019). Molecular Therapy-Nucleic Acids, 16, 302-312.	BI-1, anti-BACE-1<br><br>UTexas: 5′ATCCAGAGTGACGCAGCAAGCGATACTGCGTGGCTGGAGGCGGGTAGGGCCAGAGTTCTGGACACGGTGGCTTAGT3′<br>Apta-Index: 5'AGCGATACTGCGTGGCTGGAGGCGGGTAGGGCCAGAGTTC3'	UTexas added primer regions.
# 10001297<br><br>Diaz-Fernandez, A., et al. (2019). Biosensors and Bioelectronics, 128, 83-90.	PSA-1, anti-Prostate-specific antigen<br><br>UTexas: 5'AGGGTTGATAGGTTAAGAGCGGACGGTTGCGCTATATTTAACCAAAAGTCTGGATTAACACGATGTCAACTAGCTGTTGGG3'<br>Apta-Index: 5'GGACGGTTGCGCTATATTTAACCAAAAGTCTGGATTAACA3'	UTexas added primer regions.
# 10001334<br><br>Song, Z., et al. (2020). Molecules, 25(23), 5585.	CD63-1, anti- CD63 positive cells<br><br>UTexas: 5′TAGGGAAGAGAAGGACATATGATTAACACGACAGACGTTCGGAGGTCGAACCCTGACAGCGTGGGCTTGACTAGTACATGACCACTTGA3′<br>Apta-Index: 5′TAACACGACAGACGTTCGGAGGTCGAACCCTGACAGCGTGGG3'	UTexas added primer regions.
# 10001339<br><br>Kohlberger, M., et al. (2020). PLoS One, 15(11), e0241560.	C7, anti-Rituximab, anti-CD20 IgG1 antibody<br><br>UTexas: 5´ATACCAGCTTATTCAATTGGCCATTGTGGACTTCTTTGGGTAATTCAGGGGCTCGATTAGATAGTAAGTGCAATCT3´<br>Apta-Index: 5′GGCCATTGTGGACTTCTTTGGGTAATTCAGGGGCTCGATT3′	UTexas added primer regions.
# 10001343<br><br>Kohlberger, M., et al. (2020). PloS one, 15(11), e0241560.	C10, anti-Rituximab, anti-CD20 IgG1 antibody<br><br>UTexas: 5´ATACCAGCTTATTCAATTACTTCGGCTAGTTAGGGGGTAGTTTAGATCGTCTCTACATAGATAGTAAGTGCAATCT3´<br>Apta-Index: 5′ACTTCGGCTAGTTAGGGGGTAGTTTAGATCGTCTCTACAT3′	UTexas added primer regions.

# 10001348<br><br>Chinnappan, R., et al. (2021). Talanta, 224, 121818.	BC1, anti- Beta-crosslap (BC)<br><br>`UTexas:     5'ATACCAGCTTATTCAATTATGACGGGGGTCTAGGCAAGTAATAACGGGGGCAAGCTTTTCTATCTCGTTCTAGGGTAAGATAGTAAGTGCAATCT3'`<br>`Apta-Index:                  5'ATGACGGGGGTCTAGGCAAGTAATAACGGGGGCAAGCTTTTCTATCTCGTTCTAGGGTA3'`	UTexas added primer regions.
# 10001349<br><br>Zhu, C., et al. (2021). Talanta, 223(Pt 1), 121690.	T-2, anti- Thyroglobulin (Tg)<br><br>`UTexas:     5'CCTAACCGATATCACACTCACCGCGTGAGCGGGGAGGCGATGCCCAGGCTAACTTGACTCAGTTGGTCGTCATTGGAGTATC3'`<br>`Apta-Index: 5'--------------------CGCGTGAGCGGGGAGGCGATGCCCAGGCTAACTTGACTCA---------------------3'`	Apta-Index deleted the primer regions.
# 10001358<br><br>Yu, Q., et al. (2021). Journal of fish diseases, 44(1), 33–44.	GVI-7, anti-Grass carp reovirus (GCRV)-infected CIK cells<br><br>`UTexas:     5'GACGCTTACTCAGGTGTGACTCGTGAACCCACCTCAGGGCATCTTACATTTCTTCTAAGTTGTTACCATGTTTCGAAGGACGCAGATGAAGTCTC3'`<br>`Apta-Index:   5'GTCTGAAGTAGACGCAGGAGTGAACCCACCTCAGGGCATCTTACATTTCTTCTAAGTTGTTACCATGTTTAGTCACACCTGAGTAAGCGT3'`	Apta-Index added incorrect primer regions (and paper has entirely different forward primer in folded aptamer, Fig 2).
# 10001372<br><br>Wu, H., et al. (2021). ACS omega, 6(5), 3771–3779.	HPA-2, anti-Helicobacter pylori<br><br>`UTexas:     5'AAGGAGCAGCGTGGAGGTTACCAGGAGGACCCTATTCTCGTGTATCGACGAGATCCAGTGACCACGACGACACACCCTAA3'`<br>`Apta-Index: 5'--------------------CCAGGAGGACCCTATTCTCGTGTATCGACGAGATCCAGTG--------------------3'`	Apta-Index deleted the primer regions.
# 10001395<br><br>Lorenzo-Gómez, R., et al. (2022). Analytica chimica acta,	D1, anti- 16-amino acid peptide from collagen XIα1<br><br>`UTexas:     5'ATACCAGCTTATTCAATTTTTTTGGTTGACGGCAGTCGGCGGTATGCGCATATCGTGTTGGTAACAATCGTAATCAGTTAG3'`<br>`Apta-Index:                       5'GGTTGACGGCAGTCGGCGGTATGCGCATATCGTGTTGGTA3'`	UTexas added primer regions.

1189, 339206.		
# 10000117<br><br>Xu, W., & Ellington, A. D. (1996). PNAS 93(15), 7475–748	C2, anti-HIV-1 Rev<br><br>UTexas:<br>5'UCUAAUACGACUCACUAUAGGGAGAGACAAGCUUGGGUCUCGACCUCGCGCGAGGAGGGUGGAGGGUCGUAGAGCGCGUAAGAAGAGAAAGAGAAGUUAAUUAAGGAUCCUCAC3'<br>Apta-Index: 5'GGAGGUCGACCUCGCGCGAGGAGGGUGGAGGGUCGUAGAGCGCGUAGGAGG3'	UTexas added primer regions rather than motif regions.
# 10000358<br><br>Proske, D., et al. (2002). The Journal of biological chemistry, 277(13), 11416–11422.	DP3, anti-Neuropeptide Y<br><br>UTexas: 5'UCGGAGAAAGGGAAGCUUGAGCAGCAGGAGGGCCGGCGUUAGGGUUAGCGAGCCGAUUGAAAGAAGAAGGAACGAGCGUACGGAUCCGAUC3'<br>Apta-Index: 5'-GGGAGAAAGGGAAGCUUGAGCAGCAGGAGGGCCGGCGUUAGGGUUAGCGAGCCGAUUGAAAGAAGAAGGAACGAGCGUACGGAUCCGAUC3'	Apta-Index deleted one nt and inserted an extra G.
# 10000392<br><br>Pileur, F., et al. (2003). Nucleic acids research, 31(19), 5776–5788.	VI-2, anti-Ribonuclease H1<br><br>UTexas: 5'GCCTGTTGTGAGCCTCCTCTCGAACGGTCGCTCCGTGTGGCTTGGGTTGGGTGTGGCAGTGACTTGAGCGTTTATTCTTGTCTCCC3'<br>Apta-Index: 5'CGGTCGCTCCGTGTGGCTTGGGTTGGGTGTGGCAGTGAC3'	UTexas added primer regions.
# 10000645<br><br>Tang, Z., et al. (2007). Analytical chemistry, 79(13), 4900–4907.	TD05, anti-Ramos cells<br><br>UTexas: 5'AAGGAGCAGCGTGGAGGATAAACACCGTGGAGGATAGTTCGGTGGCTGTTCAGGGTCTCCTCCCGGTGTTAGGGTGTGTCGTCGTGGT3'<br>Apta-Index: 5'AACACCGTGGAGGATAGTTCGGTGGCTGTTCAGGGTCTCCTCCCGGTG3'	UTexas added primer regions.

# 10000697<br><br>Li, M., et al. (2008). Journal of the American Chemical Society, 130(38), 12636–12638.	85A, anti-Fibrinogen<br><br>UTexas: 5′CCTTCGTTGTCTGCCTTCGTAGCGGATCGAATTACGCGTTAACGGCAACCGATAACGGGACCGATTGCACACCCTTCAGAATTCGCACCA3′<br>Apta-Index: 5'CCTTCGTTGTCTGCCTTCGTAGGACCGCAGACATCGACGCAGGGAAATTCCGCAAGTCCAGCCAAATGCCACCCTTCAGAATTCGCACCA3'	Apta-Index reported a different random region and the correct primer regions.
# 10000800<br><br>Savory, N., et al. (2010). Biosensors & bioelectronics, 26(4), 1386–1391.	PSap4#5, anti-Prostate specific antigen (PSA)<br><br>UTexas: 5′CATGCTTACCTATAGTGAACTTTATTAGCCTCCCGGAAGAGCACCTCTTTCATGCTTACCTATAGTGAAC3′<br>Apta-Index: 5'TTTTTAATTAAAGCTCGCCATCAAATAGCTTT3'	Apta-Index reported the ΔPSap4#5 aptamer as PSap4#5
# 10000885<br><br>Song, KM, Jet al. Anal Bioanal Chem. 2012;402(6): 2153-2161.	AMP18, anti-Ampicillin<br><br>UTexas: 5′CACCTAATACGACTCACTATAGCGGATCCGA-TTAGTTGGGGTTCAGTTGGCTGGCTCGAACAAGCTTGC3′<br>Apta-Index: 5'TTTAGTTGGGGTTCAGTTG3'	UTexas added primer regions and deleted a T nucleotide.
# 10000899<br><br>Woo, H. M., et al. (2013). Antiviral research, 100(2), 337–345.	NS1 aptamer, anti-Influenza virus non-structural protein 1 (NS1) protein<br><br>UTexas: 5'GCAATGGTACGGTACTTCCGCGGTCCGGGGTGGGTGGGTGGTGGGGGGTGCGGG----------CAAAAGTGCACGCTACTTTGCTAA3'<br>Apta-Index: 5'GCAATGGTACGGTACTTCCGCGGTCCGGGGTGGGTGGGTGGTGGGGGGTGCGGGGGGGCGGCCGCAAAAGTGCACGCTACTTTGCTAA3'	UTexas deleted 10 nucleotides.

# 10000918<br><br>Eissa, S., et al. (2013). Analytical chemistry, 85(24), 11794–11801.	OA34, anti-Okadaic acid (OA)<br><br>UTexas: 5'ATACCAGCTTATTCAATTGGTCACCAACAACAGGGAGCGCTACGCGAAGGGTCAATGTGACGTCATGCGGATGTGTGGAGATAGTAAGTGCAATCT3'<br>Apta-Index: 5'GGTCACCAACAACAGGGAGCGCTACGCGAAGGGTCAATGTGACGTCATGCGGATGTGTGG3'	UTexas added primer regions.
# 10000968<br><br>Yang, M., et al. (2013). Sensors (Basel, Switzerland), 13(5), 6865–6881.	Apt22, anti- Salmonella Paratyphi A<br><br>UTexas: 5'GAATTCAGTCGGACAGCGATGGACGAATATCGTCTCCCAGTGAATTCAGTCGGACAGCGGATGGACGAATATCGTCTCCC3'<br>Apta-Index: 5'ATGGACGAATATCGTCTCCCAGTGAATTCAGTCGGACAGCG3'	Sequence from the paper is unclear (i.e., unclear if primer regions are added or not).
# 10000981<br><br>Xu, D., et al. (2014). Nucleic acid therapeutics, 24(3), 226–238.	AptER-1, anti-Estrogen receptor alpha (ERα)<br><br>UTexas: 5’GGGCAGAGGCACCGCGAACAAAACGCAAGACAGAGUGCCGACAAGAGCACUACAAGCUUCUGCCC3’<br>Apta-Index: 5’GGGCAGACGCACCGCGAACAAAACGCAAGACAGAGUGCCGACAAGAGCACUACAAGCUUCUGCCC3’	Apta-Index introduced a mutation (changed C to G)
# 10000795<br><br>Chang, T. W., et al. (2010). Biochemical and biophysical research communications, 396(4), 854–860.	S132B-C22, anti-Light chain of type A botulinum neurotoxin (BoNT/A) (LCA)<br><br>UTexas: 5’GGGAGGAGGAGAGAUGUGAACUUGACAGCGUGCCUAGAAGUCCAAGCUUAAAUAACCACGCUCGACAAGCAGAAACUCUACACUGGACUGGCG3’<br>Apta-Index: 5'GGGAGGAGGAGAGATGTGAACTTGACAGCGUGCCUAGAAGUCCAAGCUUAAAUAACCACGCUCGACAAGCAGAAACTCTACACTGGACTGGCG3'	Apta-Index included T’s, instead of U’s for an RNA Aptamer

# 10001357<br><br>Yu, Q., et a. (2021). Journal of fish diseases, 44(1), 33–44.	GVI-1, anti-Grass carp reovirus (GCRV)-infected CIK cells<br><br>UTexas: 5'GACGCTTACTCAGGTGTGACTCGGGGTGTAGCTCGTTATGATTCGGACAAGACTTACCTTGCGCCTCTGGGATCGAAGGACGCAGATGAAGTCTC3'<br>Apta-Index: 5'GTCTGAAGTAGACGCAGGAGGGGTGTAGCTCGTTATGATTCGGACAAGACTTACCTTGCGCCTCTGGGATAGTCACACCTGAGTAAGCGT3'	Apta-Index recorded incorrect primer regions.

Table S3: Comparison of Aptamer Databases: Examination of those sequences that differed.

When comparing the established Apta-Index to the UTexas Aptamer Database, we found 90 aptamer sequences in common between the two. Of the 27 that were different, we examined the source of the differences to build our internal training practices when extracting sequence information from the literature.
